# New freshwater mussel taxa discoveries clarify biogeographic division of Southeast Asia

**DOI:** 10.1038/s41598-020-63612-5

**Published:** 2020-04-20

**Authors:** Ivan N. Bolotov, Ekaterina S. Konopleva, Ilya V. Vikhrev, Mikhail Yu. Gofarov, Manuel Lopes-Lima, Arthur E. Bogan, Zau Lunn, Nyein Chan, Than Win, Olga V. Aksenova, Alena A. Tomilova, Kitti Tanmuangpak, Sakboworn Tumpeesuwan, Alexander V. Kondakov

**Affiliations:** 1 Northern Arctic Federal University, Northern Dvina Emb. 17, 163002 Arkhangelsk, Russian Federation; 2 Federal Center for Integrated Arctic Research, Russian Academy of Sciences, Northern Dvina Emb. 23, 163000 Arkhangelsk, Russian Federation; 3 CIBIO/InBIO – Research Center in Biodiversity and Genetic Resources, University of Porto, Campus Agrário de Vairão, Rua Padre Armando Quintas 7, 4485-661 Vairão, Portugal; 4 CIIMAR/CIMAR – Interdisciplinary Centre of Marine and Environmental Research, University of Porto, Terminal de Cruzeiros do Porto de Leixões, Avenida General Norton de Matos, S/N, 4450-208 Matosinhos, Portugal; 5 SSC/IUCN – Mollusc Specialist Group, Species Survival Commission, International Union for Conservation of Nature, c/o The David Attenborough Building, Pembroke Street, CB2 3QZ Cambridge, United Kingdom; 6 North Carolina Museum of Natural Sciences, 11 West Jones St., Raleigh, NC 27601 USA; 7 Fauna & Flora International – Myanmar Programme, Yangon, Myanmar; 8 Department of Zoology, Dawei University, Dawei, Tanintharyi Region Myanmar; 9 Department of Science, Faculty of Science and Technology, Loei Rajabhat University, Loei, Thailand; 10 Department of Biology, Faculty of Science, Mahasarakham University, Maha Sarakham, Thailand

**Keywords:** Biodiversity, Biogeography, Taxonomy

## Abstract

While a growing body of modern phylogenetic research reveals that the Western Indochina represents a separate biogeographic subregion having a largely endemic freshwater fauna, the boundaries of this subregion are still unclear. We use freshwater mussels (Unionidae) as a model to reconstruct spatial patterns of freshwater biogeographic divides throughout Asia. Here, we present an updated freshwater biogeographic division of mainland Southeast Asia and describe 12 species and 4 genera of freshwater mussels new to science. We show that the Isthmus of Kra represents a significant southern biogeographic barrier between freshwater mussel faunas of the Western Indochina and Sundaland subregions, while the Indian and Western Indochina subregions are separated by the Naga Hills, Chin Hills, and Rakhine Yoma mountain ranges. Our findings highlight that the freshwater bivalve fauna of Southeast Asia primarily originated within three evolutionary hotspots (Western Indochina, Sundaland, and East Asian) supplemented by ancient immigrants from the Indian Subcontinent.

## Introduction

Freshwater mussels (Unionida) are an economically and environmentally important group of aquatic animals having a broad distribution on all continents except Antarctica^1,2^. Southeast Asia houses one of the richest endemic faunas of freshwater mussels globally^3–7^. Unfortunately, freshwater mussels are among the most endangered animal groups at the global scale, with numerous local extinctions triggered by multiple anthropogenic impacts and climate changes^8–11^. Human-mediated degradation of natural habitats, e.g. water pollution, river damming, and irrigation practices, appears to be the most influential factor causing the decline and local extinctions of freshwater mussels^11–13^. It was shown that even a prehistoric decline in freshwater mussels corresponds to the early development of agricultural techniques^14^. Alien species may represent a significant threat to native freshwater mussel assemblages in Southeast Asia^13^ and other regions^15^. For example, the tropical lineage of *Sinanodonta woodiana* (Lea, 1834) is widely spread throughout Malaysia, the Indonesian Archipelago, and the Philippines^16–18^, while the temperate lineage of this taxon was found in Myanmar^19^.

Recent advances in mitogenomic^20^ and multi-locus nuclear^21^ phylogenetic modeling reveal that two widespread Southeast Asian subfamilies of the Unionidae, i.e. Pseudodontinae^22^ and Rectidentinae^22,23^, represent tribes within the monophyletic Gonideinae. The genus- and species-level taxonomy of freshwater mussels in Southeast Asia is still poorly known^3^, but several integrative studies performed in Myanmar, Thailand, Laos, and Malaysia have recently improved our knowledge about the diversity and biogeographic patterns in the region. These studies found that freshwater mussel faunas of Myanmar, from the Ayeyarwady to Salween and Dawei basins, could be considered as a separate and distinct freshwater biogeographic subregion from the Indian and Sundaland subregions^4–7,22,24^. This subregion harbors a species-rich, largely endemic fauna of freshwater mussels, with one endemic tribe, Leoparreysiini Vikhrev, Bolotov & Kondakov, 2017^4,6^. Broad-scale phylogenetic research revealed that the Unionidae fauna of Malaysia has 9 native species^16^, all of which are representatives of Sundaland genera^4,22^.

Our knowledge of freshwater mussels from the Mekong Basin and coastal rivers of Thailand and Cambodia is also far from being complete. However, there have been recent advances in the research on the freshwater mussel diversity in this region. It is now known that the genus *Contradens* Haas, 1911 in Thailand contains at least three allopatric species, one of which is widespread throughout the Chao Phraya, Mae Klong, and Bang Pakong basins corresponding to the former Siam Paleo-River system^25^. In contrast, two other species share restricted ranges in the northeastern part of the Khorat Plateau^25^. Two monotypic genera from the Mekong Basin, i.e. *Unionetta* Haas, 1955 and *Harmandia* Rochebrune, 1881, were found to be members of the tribe Indochinellini Bolotov *et al*., 2018^26^. It was revealed recently that *Scabies* Haas, 1911, another member of the Indochinellini, contains at least eight valid species from Thailand, while *S. songkramensis* Kongim & Panha, 2015 takes a distant phylogenetic position leading to non-monophyly of this genus in its current understanding^25,27^. The genus *Ensidens* Frierson, 1911 in Thailand includes two large clades, one of which contains four species and corresponds to tributaries of the Middle Mekong drainage^28^. The second clade comprises two species: one species primarily from the Siam Paleo-River system, and another species from the Lower Mekong and Bang Pakong River basins.

The present study aims to update the freshwater biogeographic divisions of mainland Southeast Asia using freshwater mussels (Unionidae) as a model group. Based on the results of a broad-scale field survey throughout Myanmar, Thailand, and northern Laos, we clarify the western and southern boundaries of the Western Indochina Subregion. During this extensive assessment, we discovered several novel genera and species of freshwater mussels that are described here to improve our current understanding of the Unionidae systematics in Southeast Asia. Finally, we show that the Isthmus of Kra is a significant biogeographic barrier separating freshwater mussel faunas of the Western Indochina and Sundaland subregions.

## Results

### New Unionidae genera and species from Southeast Asia

**Figure 1:**
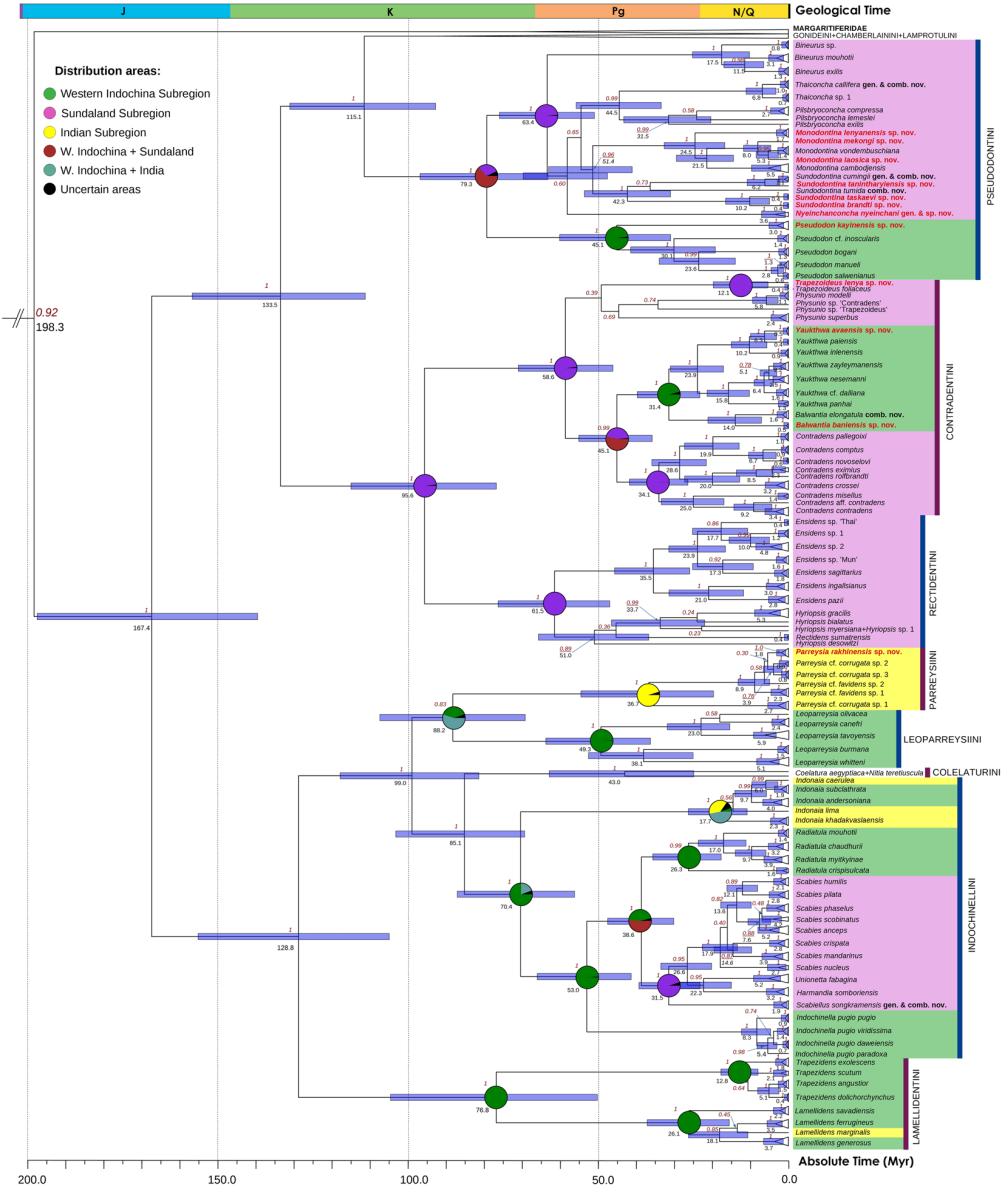
Time-calibrated multi-locus phylogeny of the Unionidae based on the complete data set of mitochondrial and nuclear sequences (five partitions: three codons of *COI* + *16* *S rRNA* + *28* *S rRNA*). Red numbers near nodes are BPP of BEAST v2.6.1. Black numbers near nodes are the node ages. Node bars are 95% HPD of the divergence time. Age reconstructions for weakly supported nodes (BPP < 0.75) are omitted. Pie charts at nodes indicate the probabilities of certain ancestral areas for clades of interest with respect to combined results under two different statistical modeling approaches (S-DIVA and Bayesian MCMC analysis). New generic and species names are colored red. Outgroup and non-target clades are collapsed. Stratigraphic chart according to the International Commission on Stratigraphy, 2019.

**Figure 2:**
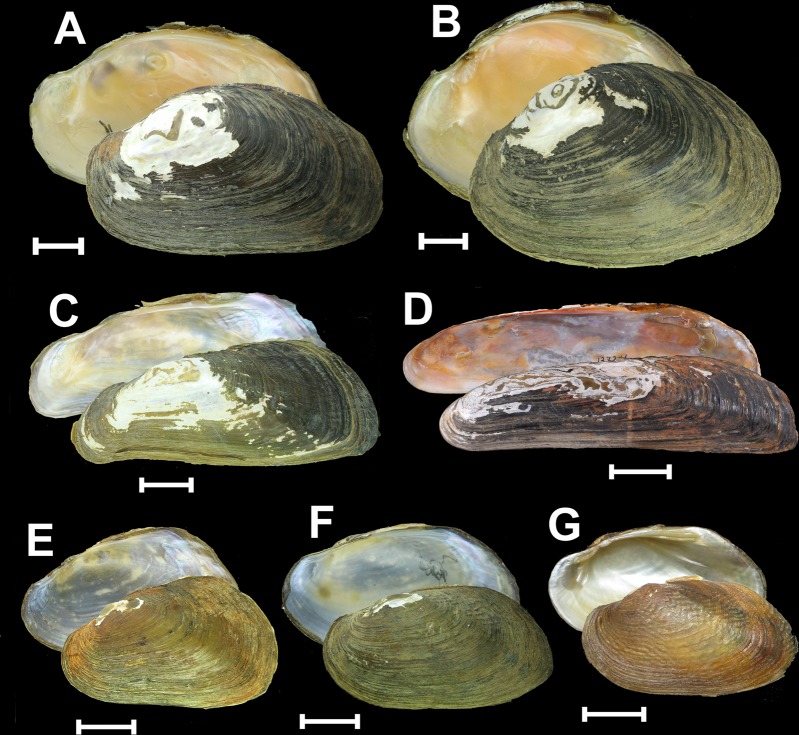
Shells of the Parreysiini, Indochinellini, Contradentini, and Rectidentini from Southeast Asia. (**A**) *Parreysia rakhinensis* **sp. nov**., Kyeintali Stream, Rakhine Coast, Myanmar (holotype RMBH biv652_1). (**B**) *Parreysia rakhinensis* **sp. nov**., Ann River, Rakhine Coast, Myanmar (paratype RMBH biv659_3). (**C**) *Balwantia baniensis* **sp. nov**., Bani River, Ayeyarwady Basin, Myanmar (holotype RMBH 666_2). (**D**) *Balwantia soleniformis* (Benson, 1836) **comb. rev**., Brahmaputra River, India (specimen USNM 127246). (**E**) *Trapezoideus lenya* **sp. nov**., 14th Mile Stream, Lenya Basin, southeastern Myanmar (holotype RMBH biv629_2). (**F**) *Yaukthwa avaensis* **sp. nov**., unnamed small stream, a tributary of the Ayeyarwady River, Myanmar (holotype RMBH biv680_3). (**G**) *Scabiellus songkramensis* (Kongim & Panha, 2015) **gen. & comb. nov**., Songkhram River, Mekong Basin, Thailand (topotype, collection of S. Tumpeesuwan, Mahasarakham University). Scale bars = 1 cm [A-C, E-G] and 3 cm [D]. Photos: Ekaterina S. Konopleva [A-C, E, F], Ellen Strong [D], and Benchawan Nahok [G].

**Figure 3:**
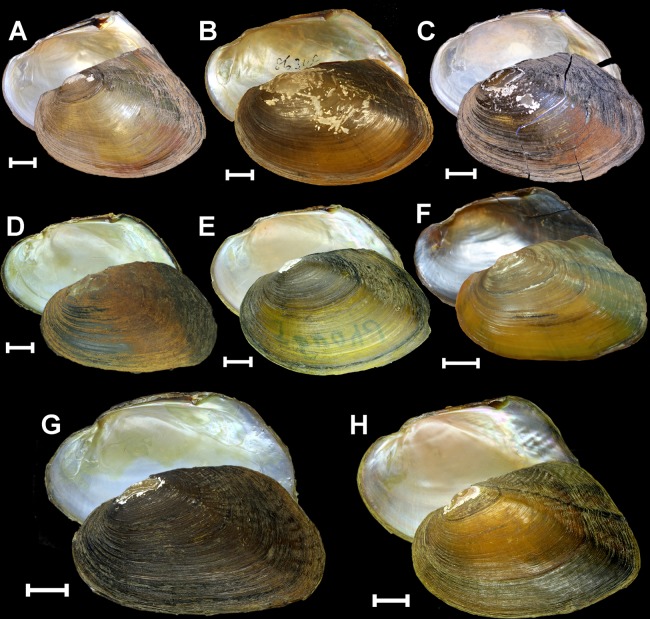
Shells of the Pseudodontini from Southeast Asia. (**A**) *Monodontina cambodjensis* (Petit de la Saussaye, 1865), Pursat River, Mekong Basin, Cambodia (specimen UMMZ 304350). (**B**) *Monodontina vondembuschiana* (Lea, 1840), Java (holotype USNM 86348). (**C**) *Monodontina laosica* **sp. nov**., Houai Pin Stream, a tributary of the Vang Ngao River, Mekong Basin, southern Laos (holotype UMMZ 304650). (**D**) *Monodontina lenyanensis* **sp. nov**., 14 Mile Stream, Lenya Basin, Myanmar (holotype RMBH biv628_2). (**E**) *Monodontina mekongi* **sp. nov**., headwater of the Phong River, Mekong Basin, Thailand (holotype RMBH biv122). (**F**) *Nyeinchanconcha nyeinchani* **gen. & sp. nov**., small stream arising at cave near Ban Kouanphavang, Mekong Basin, central Laos (holotype NCSM 84884). (**G**) *Pseudodon kayinensis* **sp. nov**., Winyaw River, Ataran Basin, southeastern Myanmar (holotype RMBH biv618_1). (**H**) *Pseudodon salwenianus* (Gould, 1844), unnamed stream, Salween Basin, Myanmar (a topotype specimen RMBH biv639_3). Scale bars = 1 cm. Photos: Taehwan Lee [A, C], Ilya V. Vikhrev [B], Ekaterina S. Konopleva [D, E, G, H], and Jamie M. Smith [F].

**Figure 4:**
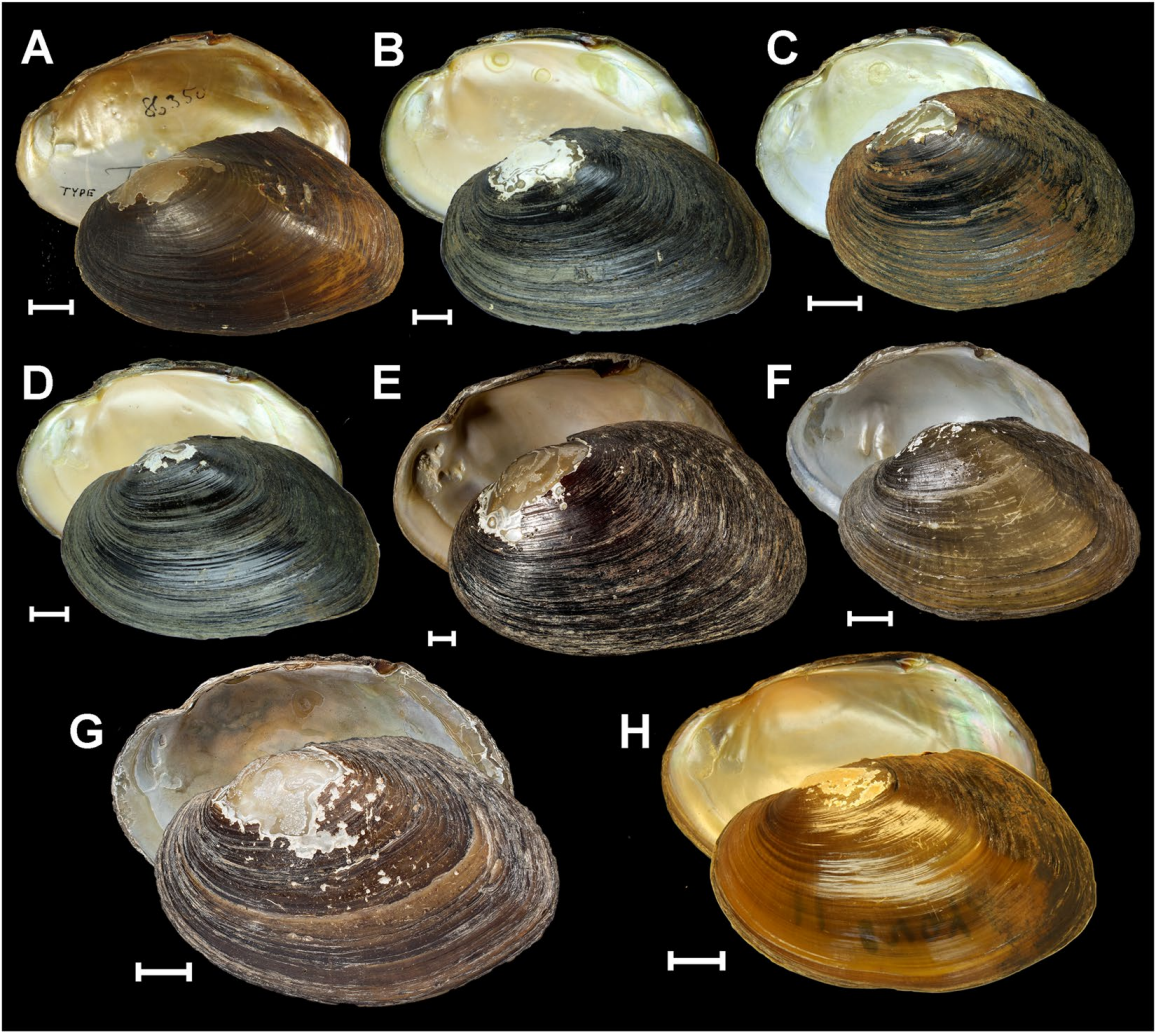
Shells of the Pseudodontini from Southeast Asia. (**A**) *Sundadontina cumingii* (Lea, 1850) **gen. & comb. nov**., Malacca (holotype USNM 86350). (**B**) *Sundadontina brandti* **sp. nov**., headwater of the Mun River, Mekong Basin, Thailand (holotype RMBH biv475_2). (**C**) *Sundadontina tanintharyiensis* **sp. nov**., Chaung Nauk Pyan Stream, Lenya Basin, Myanmar (holotype RMBH biv643_4). (**D**) *Sundadontina taskaevi* **sp. nov**., headwater of the Mun River, Mekong Basin, Thailand (holotype RMBH biv475_1). (**E**) *Sundadontina moreleti* (Crosse & Fischer, 1876) **comb. nov**., Mekong Basin, Cambodia (syntype MNHN-IM-2000–34623). (**F**) *Sundadontina tumida* (Morelet, 1866) **comb. nov**., Cambodia (holotype NHMUK 93-2-4-1734). (**G**) *Thaiconcha callifera* (Martens, 1860) **gen. & comb. nov**., Siam (holotype NHMUK 1859-8-1-20). (**H**) *Thaiconcha callifera* (Martens, 1860) **gen. & comb. nov**., (a topotype specimen RMBH biv120_11). Scale bars = 1 cm. Photos: Ilya V. Vikhrev [A], Ekaterina S. Konopleva [B-D, H], Kevin Webb (NHMUK Photographic Unit) [F, G], and Manuel Caballer (2018 MNHN Project: RECOLNAT No. ANR-11-INBS-0004) [E].

**Figure 5:**
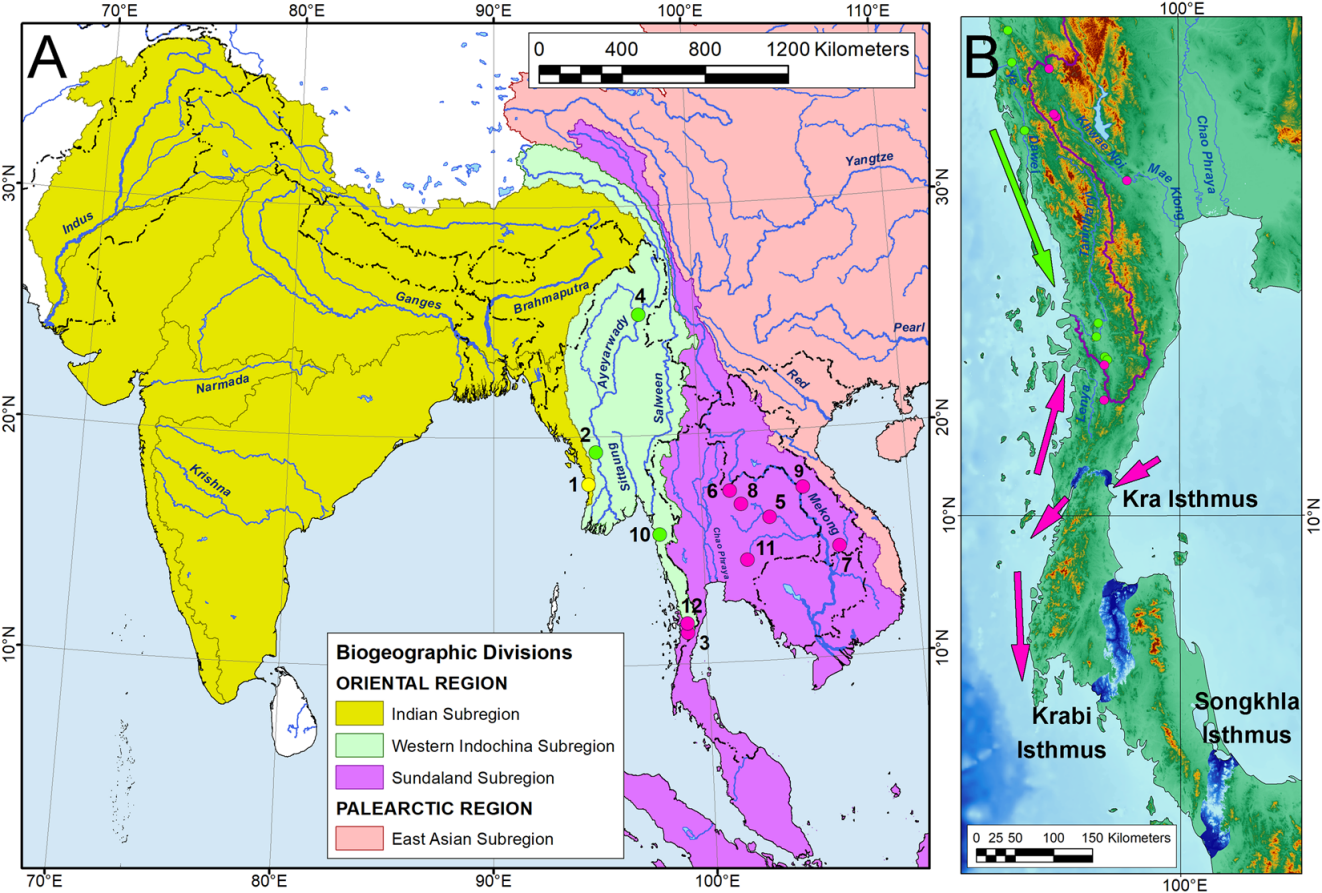
Updated freshwater biogeographic division of the mainland Southeast Asia based on freshwater mussel phylogenetics. (**A**) Freshwater biogeographic division of the mainland Southeast Asia. Color circles indicate the localities of taxa having biogeographic affinities to the Indian (yellow), Western Indochina (green), and Sundaland (pink) faunas. Type localities of new freshwater mussel species and occurrences of two cryptic *Ensidens* lineages are numbered as follows: *Parreysia rakhinensis* **sp. nov**. (1), *Balwantia baniensis* **sp. nov**. (2), *Trapezoideus lenya* **sp. nov**. and *Monodontina lenyanensis* **sp. nov**. (3), *Yaukthwa avaensis* **sp. nov**. (4), *Ensidens* sp. ‘Mun’ (5), *Ensidens* sp. ‘Thai’ (6), *Monodontina laosica* **sp. nov**. (7), *M. mekongi* **sp. nov**. (8), *Nyeinchanconcha nyeinchani* **gen. & sp. nov**. (9), *Pseudodon kayinensis* **sp. nov**. (10), *Sundadontina brandti* **sp. nov**. and *S*. *taskaevi* **sp. nov**. (11), and *S. tanintharyiensis* **sp. nov**. (12). (**B**) Boundary between the Western Indochina and Sundaland freshwater subregions at the southern margin of the Kra Isthmus (Tanintharyi – Lenya drainage divide). Violet line indicates the boundary between freshwater subregions based on drainage divides of the corresponding river basins. Green circles indicate records of the Western Indochina fauna representatives: *Leoparreysia tavoyensis*, *Trapezidens scutum*, *T. exolescens*^4,24^, *Indochinella pugio daweiensis*^6^. Pink circles indicate records of the Sundaland fauna representatives: *Trapezoideus foliaceus*^7^, *Trapezoideus lenya* **sp. nov**., *Monodontina lenyanensis* **sp. nov**., and *Sundadontina tanintharyiensis* **sp. nov**. All freshwater mussel taxa in Malaysia are members of the Sundaland fauna^16,22^. Arrows indicate putative ancient (pre-Pleistocene) dispersal routes of the Western Indochina (green) and Sundaland (pink) Unionidae species around the Isthmus of Kra and surrounding areas inferred from our statistical biogeographic analyses and distribution data (Supplementary Tables 1 and 3). Blue gradient shading indicates the putative ancient seaways crossing the Thai-Malay Peninsula based on the ArcGIS modeling (hydrologically conditioned DEM with elevation levels < 120 m) and published data^36^. The map was created using ESRI ArcGIS 10 software (https://www.esri.com/arcgis); the topographic base of the map was created with Natural Earth Free Vector and Raster Map Data (https://www.naturalearthdata.com), Global Self-consistent Hierarchical High-resolution Geography, GSHHG v2.3.7 (https://www.soest.hawaii.edu/wessel/gshhg), HydroSHEDS (https://www.hydrosheds.org)^81^, The General Bathymetric Chart of the Oceans, GEBCO (https://www.gebco.net), and Vector Map (VMap) Level 0 (http://gis-lab.info/qa/vmap0-eng.html) (Maps: Mikhail Yu. Gofarov).

Our multi-locus phylogenies were constructed using BEAST v2.6.1, MrBayes v3.2.6 and IQ-TREE v1.6.11 based on the mitochondrial *cytochrome c oxidase subunit I* (*COI*), *small ribosomal RNA* (*16* *S rRNA*), and the nuclear *large ribosomal RNA* (*28* *S rRNA*) gene fragments. These analyses returned well-resolved consensus phylogenies having a similar topology (Fig. 1 and Supplementary Fig. 1). We found that available freshwater mussel taxa from Southeast Asia cluster to at least 25 genera, four of which are new to science and are described here. The novel genera represent distant monotypic lineages (*Scabiellus* **gen. nov**. and *Nyeinchanconcha* **gen. nov**.) and well-supported clades with several species (*Sundadontina* **gen. nov**. and *Thaiconcha* **gen. nov**.). An integrative species delimitation analysis indicates that our dataset contains 12 species that do not have available names and can be considered new to science (Figs. 1–5, Tables 1–3, Supplementary Tables 1–2). Each novel species can be distinguished from its sister taxa by conchological and molecular characters. A description of each new species is presented below. Mean shell parameters for the type series of new species are presented in Table 1. Two more unnamed species-level lineages, i.e. *Ensidens* sp. ‘Mun’ and *Ensidens* sp. ‘Thai’ (Fig. 1), appear to represent cryptic species and require separate research.

**Table 1:** Mean shell parameters (mm) for the type series of new freshwater mussel species (Unionidae) from Southeast Asia.

Species	Shell Length (SL)		Shell Height (SH)		Shell Width (SW)	
	Mean ± s.e.m.	Min-max	Mean ± s.e.m.	Min-max	Mean ± s.e.m.	Min-max
*Parreysia rakhinensis* **sp. nov**. (*N* = 26)	45.9 ± 1.7	29.3–62.3	28.8 ± 1.1	18.5–41.7	17.3 ± 0.7	10.3–24.2
*Balwantia baniensis* **sp. nov**. (*N* = 5)	54.1 ± 2.9	46.1–61.4	22.7 ± 1.2	19.5–26.3	14.2 ± 0.9	12.7–16.7
*Trapezoideus lenya* **sp. nov**. (*N* = 4)	35.2 ± 0.8	33.1–36.3	20.6 ± 0.6	19.1–21.4	11.3 ± 0.6	10.2–12.6
*Yaukthwa avaensis* **sp. nov**. (*N* = 10)	37.8 ± 2.8	24.7–45.6	21.0 ± 1.4	14.1–25.1	14.4 ± 1.2	8.7–17.0
*Monodontina laosica* **sp. nov**. (*N* = 1)	n/a	61.4	n/a	41.4	n/a	19.0
*M. lenyanensis* **sp. nov**. (*N* = 10)	51.1 ± 3.6	31.2–63.4	32.4 ± 2.2	20.3–40.1	17.5 ± 1.4	11.0–23.7
*M. mekongi* **sp. nov**. (*N* = 1)	n/a	65.7	n/a	42.2	n/a	20.3
*Pseudodon kayinensis* **sp. nov**. (*N* = 11)	52.6 ± 3.4	37.8–71.0	30.9 ± 1.9	22.4–42.5	15.9 ± 1.1	10.3–21.2
*Sundadontina brandti* **sp. nov**. (*N* = 3)	78.4 ± 5.0	71.3–85.4	49.3 ± 3.6	43.8–53.7	26.3 ± 1.4	24.1–27.7
*S. tanintharyiensis* **sp. nov**. (*N* = 3)	52.5 ± 8.3	39.2–61.2	37.7 ± 6.6	27.2–45.1	20.4 ± 3.5	14.7–23.2
*S. taskaevi* **sp. nov**. (*N* = 2)	71.4 ± 11.4	60.0–82.7	47.5 ± 4.7	42.8–52.2	26.0 ± 3.3	22.6–29.3
*Nyeinchanconcha nyeinchani* **gen. & sp. nov**. (*N* = 3)	48.2 ± 9.5	33.7–59.5	26.6 ± 7.8	15.1–37.1	15.0 ± 4.8	7.9–21.5

n/a – not available.

**Table 2:** Molecular diagnoses of the new freshwater mussel species (Unionidae) from Southeast Asia.

New species	Mean *COI p*-distance from the most closely related species (%)	Most closely related species	Fixed unique nucleotide differences based on the sequence alignment of congeners		
			*COI*	*16* *S rRNA*	*28* *S rRNA*
*Parreysia rakhinensis* **sp. nov**.	2.12	*Parreysia* cf. *corrugat*a sp.2	29 G, 429 C, 557 A	n/a	n/a
*Balwantia baniensis* **sp. nov**.	5.05	*Balwantia elongatula* **comb. nov**.	15 T, 17 G, 35 C, 101 G, 104 C, 159 C, 167 G, 170 A, 173 A, 182 G, 194 T, 207 T, 236 C, 242 A, 243 C, 248 T, 263 A, 290 A, 296 G, 338 A, 347 A, 353 G, 404 A, 443 T, 470 T, 506 T, 537 C, 542 G, 579 C, 587 T, 626 T, 635 C	192 T, 253 C, 263 G, 313 T, 464 T, 469 T	212 C, 497 T, 609 C
*Trapezoideus lenya* **sp. nov**.	4.46	*Trapezoideus foliaceus*	6 T, 14 G, 35 G, 38 G, 86 A, 92 G, 93 T, 98 C, 170 T, 209 A, 317 G, 353 T, 365 G, 383 T, 392 C, 401 A, 402 T, 404 A, 413 T, 461 G, 479 A, 527 G, 557 A, 584 C, 599 G, 608 T, 617 C, 629 A, 647 T, 654 C	155 T, 234 C, 252 T, 299 C, 316 A, 344 A, 355 G	n/a
*Yaukthwa avaensis* **sp. nov**.	2.90	*Yaukthwa paiensis*	365 A, 626 A	n/a	n/a
*Monodontina laosica* **sp. nov**.	2.40	*Monodontina mekongi* **sp. nov**.	89 A, 164 G, 347 A, 500 C, 539 A, 608 G	18 G, 48 C, 191 T	n/a
*Monodontina lenyanensis* **sp. nov**.	7.59	*Monodontina vondembuschiana*	86 G, 96 C, 146 A, 149 G, 197 A, 200 C, 206 A, 284 C, 287 T, 290 A, 389 A, 479 A, 480 T, 500 A, 512 A, 518 A, 521 A, 531 A, 611 C	258 C, 295 T, 334 T	528 G, 609 T, 738 C, 755 A
*Monodontina mekongi* **sp. nov**.	2.40	*Monodontina laosica* **sp. nov**.	56 A, 89 T, 257 A, 515 C, 527 G, 596 C	18 A, 48 T, 155 G	638 A, 768 A
*Nyeinchanconcha nyeinchani* **gen. & sp. nov**.	9.42	*Sundadontina brandti* **sp. nov**.	134 T, 194 C, 278 C, 299 C, 575 A	19 C, 150 C, 185 C, 196 A, 234 A, 236 C, 242 G, 296 A, 320 T, 329 T, 335 T, 338 G, 343 A, 457 T, 465 T	n/a
*Pseudodon kayinensis* **sp. nov**.	11.10	*Pseudodon cf. inoscularis*	14 A, 23 T, 53 A, 62 G, 65 C, 68 A, 83 A, 101 T, 104 C, 110 A, 131 A, 179 C, 203 C, 213 G, 224 T, 266 G, 272 A, 291 C, 311 A, 314 T, 326 A, 338 G, 344 G, 347 G, 371 A, 380 A, 437 C. 464 C, 470 G, 482 A, 491 A, 521 A, 524 A, 569 C, 587 C, 605 A, 623 T	7 C, 15 T, 25 G, 47 T, 48 C, 127 G, 147 C, 154 T, 159 G, 234 C, 240 C, 243 C, 247 T, 253 T, 293 C, 310 T, 311 C, 319 A, 320 C, 323 A, 329 T, 331 C	489 C
*Sundadontina brandti* **sp. nov**.	3.95	*Sundadontina taskaevi* **sp. nov**.	116 C, 398 C, 483 G, 518 C, 531 G, 581 A	18 C, 49 C, 371 C, 445 T	n/a
*Sundadontina tanintharyiensis* **sp. nov**.	2.46	*Sundadontina cumingii* **gen. & comb. nov**.	86 T, 206 G, 287 A, 338 G, 485 C	12 C, 14 T, 15 T, 17 T, 20 C, 47 T, 48 C, 162 T, 168 A, 172 G, 193 C, 194 A, 234 A, 243 G, 248 C, 253 G, 263 T, 318 G, 322 A, 323 T, 329 A, 335 T, 344 T, 375 T, 440 C, 475 A	n/a
*Sundadontina taskaevi* **sp. nov**.	3.95	*Sundadontina brandti* **sp. nov**.	149 A, 269 C, 380 A, 401 A, 440 C, 518 T, 536 A	159 T, 265 T, 342 A	n/a

n/a – not available. Del – deletion mutation.

**Table 3:** Taxonomic review of freshwater mussel genera under discussion within the boundaries of Southeast Asia (Myanmar, Thailand, Cambodia, Laos, and the Lower Mekong in Vietnam).

Genus and species	Type locality	Distribution
***Parreysia*** **Conrad, 1853**		
*P. rakhinensis* **sp. nov**.	Kyeintali Stream upstream of Ohtein village [17.9193°N, 94.5946°E], Myanmar	Coastal rivers of the Rakhine State, Myanmar (from Kyeintali to Ann)
***Scabiellus*** **gen. nov**.		
*S. songkramensis* (Kongim & Panha, 2015) **gen. & comb. nov**. [ = *Scabies songkramensis* Kongim & Panha, 2015]	Houy Plahang Stream [17.4061°N, 103.8336°E], Songkram River Basin, Thailand^30^	Songkhram and Kam river basins, and the corresponding section of the Mekong River, Thailand^30^
***Balwantia*** **Prashad, 1919 stat. rev**.		
*B. elongatula* (Bolotov *et al*., 2019) **comb. nov**. [ = *Yaukthwa elongatula* Bolotov *et al*., 2019]	Chindwin River [23.1918ºN, 94.3217ºE], Ayeyarwady Basin, Myanmar^6^	Chindwin River, Ayeyarwady Basin, Myanmar^6^
*B. baniensis* **sp. nov**.	Bani River at Bangong village [19.3247°N, 94.9839°E], Ayeyarwady Basin, Myanmar	Bani River, Myanmar
***Trapezoideus*** **Simpson, 1900**		
*T. foliaceus* (Gould, 1843) [ = *Unio foliaceus* Gould, 1843]	Tavoy [Dawei River], British Burma^7^	Mae Klong River, Thailand, and Dawei River, Myanmar^7^
*T. lenya* **sp. nov**.	14 Mile Stream [11.3508°N, 99.1093°E], Lenya Basin, Myanmar	Lenya Basin, Myanmar
***Yaukthwa*** **Konopleva** ***et al****.***, 2019**		
*Y. avaensis* **sp. nov**.	Tarkat Stream [25.2758°N, 97.2722°E], tributary of Ayeyarwady River, Myanmar	Ayeyarwady River, Myanmar
*Y. dalliana* (Frierson, 1913) [ = *Parreysia dalliana* Frierson, 1913]	Lashio River near Lashio [approx. 22.9946°N, 97.7650°E], Ayeyarwady Basin, Myanmar^7^	Upper Ayeyarwady Basin, Myanmar^7^
*Y. inlenensis* Konopleva *et al*., 2019	Mway Stream [19.7266°N, 97.0992°E], a tributary of Nam Pilu River, Salween Basin, Myanmar^7^	Lake Inle Area, Salween Basin, Myanmar^7^
*Y. nesemanni* (Konopleva, Vikhrev & Bolotov, 2017) [ = *Trapezoideus nesemanni* Konopleva, Vikhrev & Bolotov, 2017]	Thauk Ye Kupt River [19.3075°N, 96.7219°E], Sittaung Basin, Myanmar^4,7^	Sittaung Basin, Myanmar^4,7^
*Y. paiensis* Konopleva *et al*., 2019	Khong River [19.4246°N, 98.4013°E], tributary of the Pai River, Salween Basin, Thailand^7^	Pai River, Salween Basin, Thailand^7^
*Y. panhai* (Konopleva, Bolotov & Kondakov, 2017) [ = *Trapezoideus panhai* Konopleva, Bolotov & Kondakov, 2017]	Kyan Hone River [19.5059°N, 96.8280°E], Sittaung Basin, Myanmar^4,7^	Sittaung Basin, Myanmar^4,7^
*Y. peguensis (Anthony, 1865) [ = *Unio peguensis* Anthony, 1865]	Pegu, British Burmah [Bago River, Myanmar]^7^	Bago River, Myanmar^7^
*Y. zayleymanensis* (Preston, 1912) [ = *Trapezoideus foliaceus* var. *zayleymanensis* Preston, 1912]	Bhamo, Ayeyarwady River [approx. 24.2669°N, 97.2210°E]^7^	Ayeyarwady River, Myanmar^7^
***Monodontina*** **Conrad, 1853**		
*M. aeneola (Drouet & Chaper, 1892) **comb. nov**. [ = *Pseudodon aeneolus* Drouet & Chaper, 1892]	Sebruang River [approx. 0.4937°N, 111.8931°E], Kapuas Basin, western Borneo^82^	Kapuas Basin, western Borneo^82^
*M. cambodjensis* (Petit de la Saussaye, 1865) [ = *Monocondylea cambodjensis* Petit de la Saussaye, 1865; = *Monocondylus orbicularis Morelet, 1866; = *Unio subtrigonus Sowerby, 1867; = *U. vagulus Fischer, 1891; = *Pseudodon cambodjensis tenerrimus Brandt, 1974]	Battambang [approx. 13.0929°N, 103.2001°E], Mekong Basin, Cambodia^83^	Mekong Basin in Thailand and Cambodia, few rivers in Malaysia^16,31^
*M. laosica* **sp. nov**.	Houai Pin, about 300 m upstream of the mouth [14.7944°N, 106.4842°E], Mekong Basin, Laos	Mekong Basin in Laos
*M. lenyanensis* **sp. nov**.	14 mile stream [11.3508°N, 99.1093°E], Lenya River basin, Myanmar	Lenya Basin, Myanmar
*M. mekongi* **sp. nov**.	Headwater of the Phong River [16.8616°N, 101.9105°E], Mekong Basin, Thailand	Phong River, Mekong Basin, Thailand
*M. vondembuschiana* (Lea, 1840) [ = *Margaritana vondembuschiana* Lea, 1840; = *Alasmodonta crispata Mousson, 1849; = *A. zollingeri Mousson, 1849; = *Monodontina buschiana Conrad, 1853; = *Monocondyloea planulata Lea, 1859; = *M. hageni Strubell, 1897 **syn. nov**.]	Java^84^	Malaysia, Sumatra and Java
*M. walpolei (Hanley, 1871) **comb. nov**. [ = *Monocondylaea walpolei* Hanley, 1871; = *Pseudodon crassus Drouet & Chaper, 1892 **syn. nov**.]	Sarawak, Borneo (by lectotype designation)^85^	Northern Borneo^85,86^
***Nyeinchanconcha*** **gen. nov**.		
*N. nyeinchani* **gen. & sp. nov**.	Small stream arising at a cave near Ban Kouanphavang [17.4578°N, 104.9263°E], Nam Done River drainage, Mekong Basin, Laos	Mekong Basin in Laos
***Pseudodon*** **Gould, 1844**		
*P. avae* (Theobald, 1873) [ = *Monocondylaea avae* Theobald, 1873]	Mandalay, Burmah^4^	Ayeyarwady Basin, Myanmar^4^
*P. bogani* Bolotov, Kondakov & Konopleva, 2017	Kanni River [19.0545°N, 96.5131°E], Sittaung Basin, Myanmar^4^	Sittaung Basin, Myanmar^4^
*P. crebristriatus (Anthony, 1865) [ = *Monocondyloea crebristriata* Anthony, 1865; = *Pseudodon (*Trigonodon*) *crebristriatus* var. *curvata* Preston, 1912]	Pegu, British Burmah^4^	Bago Basin, Myanmar^4^
*P. inoscularis* (Gould, 1844) [ = *Anodon inoscularis* Gould, 1844]	River Salwen, Tavoy, Brit. Burmah^4^	?Dawei River, Myanmar
*P. kayinensis* **sp. nov**.	Winyaw River [15.6685°N, 97.9496°E], Ataran River basin, Myanmar	Salween Basin, Myanmar
*P. manueli* Konopleva, Kondakov & Vikrev, 2017	Pyowne Stream [18.9694°N, 96.5309°E], Sittaung Basin, Myanmar^4^	Sittaung Basin, Myanmar^4^
*P. peguensis (Anthony, 1865) [ = *Monocondyloea peguensis* Anthony, 1865]	Pegu, British Burmah^4^	Bago Basin, Myanmar^4^
*P. salwenianus* (Gould, 1844) [ = *Anodon salweniana* Gould, 1844]	Salwen River, British Burmah^4^	Salween Basin, Myanmar^4^
***Sundadontina*** **gen. nov**.		
*S. brandti* **sp. nov**.	Headwater of the Mun River [14.4138°N, 102.0821°E], Mekong Basin, Thailand	Mun River, Mekong Basin, Thailand
*S. cumingii* (Lea, 1850) **gen. & comb. nov**. [ = *Anodonta cumingii* Lea, 1850; *Pseudodus chaperi Morgan, 1885 **syn. nov**.]	Malacca^87^	Malaysia^16^
*S. harmandi (Crosse & Fischer, 1876) **comb. nov**. [ = *Pseudodon harmandi* Crosse & Fischer, 1876]	Cambodia^88^	Lower Mekong Basin in Cambodia
*S. mabilli (Rochebrune, 1881) **comb. nov**. [ = *Pseudodon mabilli* Rochebrune, 1881]	Mekong, Shigloni Breithon, Cochinchina^89^	Lower Mekong Basin in southern Vietnam
*S. moreleti Crosse & Fischer, 1876 [ = *Pseudodon moreleti* Crosse & Fischer, 1876]	Mekong, Kompang Cham Province, Cambodia^88^	Lower Mekong Basin in Cambodia
*S. tanintharyiensis* **sp. nov**.	Chaung Nauk Pyan stream [11.7620°N, 99.1124°E], Lenya River basin, Myanmar	Lenya Basin, Myanmar
*S. ponderosa (Preston, 1909) **comb. nov**. [ = *Pseudodon ponderosa* Preston, 1909]	Nan-ko, Siam [Nan River, Chao Phraya Basin, Thailand]^90^	Chao Phraya Basin, Thailand
*S. sulcatum (Rochebrune, 1881) **comb. nov**. [ = *Pseudodon sulcatum* Rochebrune, 1881]	Mouth of the Mekong River, Cochinchina^89^	Mekong Delta in southern Vietnam
*S. taskaevi* **sp. nov**.	Headwater of the Mun River [14.4138°N, 102.0821°E], Mekong Basin, Thailand	Mun River, Mekong Basin, Thailand
*S. tumida* (Morelet, 1866) **comb. nov**. [ = *Monocondylus tumidus* Morelet, 1866]	Cambodia^91^	Lower Mekong Basin in Cambodia and southern Vietnam
***Thaiconcha*** **gen. nov**.		
*T. callifera* (Martens, 1860) **gen. & comb. nov**. [ = *Anodonta callifera* Martens, 1860; *Pseudodon ellipticum* Conrad, 1865 **syn. nov**.; *P. thomsoni* Morlet, 1884 **syn. nov**.]	Siam [Thailand]^92^	Mekong Basin in Cambodia and Thailand
*T. ovalis (Morlet, 1889) **comb. nov**. [ = *Pseudodon ovalis* Morlet, 1889]	Srakeo River, Siam [Thailand]^93^	Bang Pakong Basin, Thailand

*These nominal taxa were provisionally placed in the corresponding genera or in the synonymy on the basis of conchological features alone, and they are in need of future molecular study and subsequent rearrangements.

### New freshwater mussel species distribution and their biogeographic affinities in Southeast Asia

Here, we examined freshwater mussels from several poorly known, remote basins such as the rivers of the Rakhine Coast (western Myanmar), the Tanintharyi (former Tenasserim) River, and the Lenya River (southeastern Myanmar) (Fig. 5A). We found that the rivers of the Rakhine Coast are inhabited by one species, *Parreysia rakhinensis* **sp. nov**. This is the first record of a Parreysiini member in Myanmar. The fauna of the Tanintharyi Basin includes several species belonging to endemic genera of the Western Indochina Subregion, i.e. *Trapezidens scutum* and *Leoparreysia tavoyensis*. In contrast, *Trapezoideus lenya* **sp. nov**., *Monodontina lenyanensis* **sp. nov**., and *Sundadontina tanintharyiensis* **sp. nov**. were discovered from the Lenya River basin, which is located just south of the Tanintharyi Basin (Fig. 5B). These species are members of the genera widely distributed throughout the Sundaland Subregion.

### Evolutionary biogeography and time-calibrated phylogeny

Our statistical biogeographic analyses (the combined results of S-DIVA and Bayesian MCMC runs) and time-calibrated Bayesian phylogeny reveal the high levels of endemism of freshwater mussel fauna within each subregion (Fig. 1 and Supplementary Table 3). The fauna of the Sundaland Subregion contains several endemic radiations of freshwater mussels belonging to two subfamilies, i.e. the subtribe Pilsbryoconchina, the tribe Rectidentini, the genera *Trapezoideus*, *Physunio*, *Contradens* (Gonideinae), and the so-called Mekong’s Indochinellini group (*Harmandia*, *Scabies*, *Scabiellus* **gen. nov**., and *Unionetta*) (Parreysiinae). The subtribe Pseudodontina, the genus *Yaukthwa* (Gonideinae), tribe Leoparreysiini, the genera *Radiatula*, *Indochinella*, and *Trapezidens* (Parreysiinae) are endemic clades to the Western Indochina Subregion. The fauna of the Indian Subregion contains the endemic clade Parreysiini, and shares some genus-level subclades with the Western Indochina, i.e. *Indonaia*, *Lamellidens*, and, most probably, *Balwantia*.

Our time-calibrated Bayesian phylogenetic model reveals that there were several splits between the Western Indochinese and Sundaland clades (Fig. 1). The oldest split between such groups occurred between the subtribes Pseudodontina and Pilsbryoconchina in the Late Cretaceous (mean age = 79.3 Myr, 95% HPD = 63.2–96.8 Myr). The Pseudodontina most likely diversified in the Western Indochinese Region and the Pilsbryoconchina evolved in the Sundaland Subregion (probability = 97.8% and 98.7%, respectively) (Supplementary Table 3). The Rectidentini + Contradentini clade (the former subfamily Rectidentinae^21^) appears to have evolved within the Sundaland Subregion since the Late Cretaceous (probability = 98.4%; mean age = 95.6 Myr, 95% HPD = 76.8–115.0 Myr). A colonization event of the *Yaukthwa* + *Balwantia* clade to Western Indochina occurred in the Early Eocene (mean age = 45.6 Myr, 95% HPD = 35.8–55.1 Myr) followed by an intra-area radiation (probability = 95.8%). Conversely, the Indochinellini seems to be a primary Western Indochinese clade evolving *in situ* since the Late Cretaceous (probability = 75.0%; mean age = 70.4 Myr, 95% HPD = 56.1–87.0 Myr). Divergence of Mekong’s Indochinellini clade from Western Indochinese *Radiatula* placed in the Late Eocene (probability = 50.9%; mean age = 38.6 Myr, 95% HPD = 30.1–47.5 Myr). After the vicariance event separating these taxa, each group diversified in isolation within a corresponding subregion (probability = 95–100%). Our analyses indicate that the Parreysiini clade is a group evolved within the Indian Subregion (probability = 95.2%), whereas the Leoparreysiini diversified within the Western Indochina (probability = 99.6%). The Indian and Indochinese *Indonaia* species groups most likely diverged in the mid-Miocene (mean age = 14.6 Myr, 95% HPD = 9.4–21.3 Myr) via a dispersal event from Western Indochina to India but with a rather low probability (47.0%).

Three novel species from the Lenya Basin have sister taxa in the Mekong River and smaller basins emptying into the Gulf of Thailand. These species likely separated by a series of splits occurred from the Oligocene – Miocene boundary to the Late Miocene as follows: (1) *Monodontina lenyanensis* **sp. nov**. vs *M. mekongi* **sp. nov**. + *M. vondembuschiana* + *M. laosica* **sp. nov**. + *M. cambodjensis* (mean age = 24.5 Myr, 95% HPD = 16.7–32.6 Myr); (2) *Trapezoideus lenya* **sp. nov**. vs *T. foliaceus* (mean age = 12.1 Myr, 95% HPD = 5.3–19.7 Myr); and (3) *Sundadontina tanintharyiensis* **sp. nov**. vs *S. cumingii* (mean age = 6.2 Myr, 95% HPD = 2.3–10.8 Myr). The split between *Parreysia rakhinensis* **sp. nov**. from western Myanmar and several species from India occurred in the Late Miocene (mean age = 5.5 Myr, 95% HPD = 2.9–8.1 Myr).

### **Taxonomic account**. Family Unionidae Rafinesque, 1820

Subfamily Parreysiinae Henderson, 1935

#### Tribe Parreysiini Henderson, 1935

Type genus: *Parreysia* Conrad, 1853 (by original designation)

Comments: A monotypic Indian tribe^4^.

Distribution: Indian Subregion from the Indus Basin^29^ to the coastal basins of the Rakhine State of Myanmar.

#### **Genus*Parreysia*Conrad, 1853**

Type species: *Unio multidentatus* Philippi, 1847 (by original designation)

Comments: A diverse Indian genus, in which the modern systematics and number of species are still uncertain, due to the lack of available molecular data. Several species from Western Indochina originally assigned to *Parreysia* were recently transferred to another genus, *Leoparreysia* Vikhrev, Bolotov & Aksenova, 2017, belonging to the tribe Leoparreysiini Vikhrev, Bolotov & Kondakov, 2017^4^. *Parreysia rakhinensis* **sp. nov**. is the first member of the true Parreysiini discovered in Myanmar (Table 3).

Distribution: As for the tribe.

#### ***Parreysia rakhinensis*sp. nov**

Figures 2A,B, 5A, Tables 1–2, Supplementary Table 2

Holotype: RMBH biv0652_1, MYANMAR: Kyeintali Stream upstream of Ohtein village, 17.9193°N, 94.5946°E, 04.xii.2018, Bogan, Bolotov, Vikhrev, Lopes-Lima, Nyein Chan and local villagers leg. Reference sequence numbers of the holotype are as follows: MN275091 (*COI*), MN307275 (*16* *S rRNA*), and MN307218 (*28* *S rRNA*). Shell measurements of the holotype are as follows: shell length (SL) 58.7 mm, shell height (SH) 33.7 mm, and shell width (SW) 19.6 mm.

Paratypes: MYANMAR: type locality, same collecting date, and collectors, 4 specimens (RMBH biv0652_2, biv0652_3, biv0652_4, biv0652_6), Sa Lu Stream, 18.1631°N, 94.4997°E, 04.xii.2018, 5 specimens (RMBH biv0653_1, biv0653_2, biv0653_3, biv0654_1, biv0654_2) and 24 specimens (NCSM 113365), Thandwe River near Ywar Shai village, 18.3741°N, 94.4952°E, 04.xii.2018, 4 specimens (RMBH biv0655_1, biv0655_2, biv0655_3, biv0656), Shwehle Stream, 18.6174°N, 94.3508°E, 05.xii.2018, 3 specimens (RMBH biv0657_1, biv0657_2, biv0657_3) and 22 specimens (NCSM 113366), Toungup River, 18.8439°N, 94.3447°E, 06.xii.2018, 3 specimens (RMBH biv0658_1, biv0658_2, biv0658_3) and 13 specimens (NCSM 113367), Ann River near Ann town, 19.8026°N, 94.0449°E, 07.xii.2018, 3 specimens (RMBH biv0659_1, biv0659_2, biv0659_3), tributary of the Ann River, 19.8035°N, 94.0460°E, 07.xii.2018, 3 specimens (RMBH biv0660_1, biv0660_2, biv0660_3) and 10 specimens (NCSM 113360), Bogan, Bolotov, Vikhrev, Lopes-Lima, Nyein Chan and local villagers leg.

Etymology: The new species name is derived from the Rakhine State of Myanmar, in which it is widely distributed.

Diagnosis: The new species is conchologically and genetically close to a group of *Parreysia* species from India with affinity to several nominal taxa such as *P. corrugata* and *P. favidens*. Its shell varies from ovate-rounded to ovate-elongated, rather thick, umbo slightly elevated, pseudocardinal teeth massive and usually indented, lateral teeth curved and strong, muscle attachment scars deep. The new species differs from all the congeners by fixed nucleotide substitutions in the *COI* gene fragment, while other genes from Indian *Parreysia* taxa are not available (Table 2).

Description: Medium-sized mussel: SL 29.3–62.3 mm, SH 18.5–41.7 mm, SW 10.3–24.2 mm. Shell shape variable, from ovate and strongly inflated to ovate-elongated and sub-compressed; inequilateral, rather solid. Umbo usually slightly elevated but may be much more developed at some specimens, with v-shaped sculpture visible only in small mussels due to strong erosion in old mussels. Periostracum from olive-green to brown. Nacre whitish, with bright peach or orange spot near the umbo cavity area, shining. Right valve with one curved lateral tooth and two pseudocardinal teeth, anterior tooth small and somewhat lamellar, posterior tooth massive, very indented. Left valve with two curved lateral teeth and two strongly indented pseudocardinal teeth, the anterior tooth usually higher than the posterior tooth. Anterior adductor scar ovate and deep, posterior adductor scar rounded and well-visible.

Distribution: Rivers and streams of the Rakhine Coast of Myanmar emptying into the Bay of Bengal.

#### Tribe Indochinellini Bolotov, Pfeiffer, Vikhrev & Konopleva, 2018

Type genus: *Indochinella* Bolotov, Pfeiffer, Vikhrev & Konopleva, 2018 (by original designation)

Comments: A large Oriental tribe, which contains seven genera: *Harmandia* Rochebrune, 1882, *Indochinella* Bolotov, Pfeiffer, Vikhrev & Konopleva, 2018, *Indonaia* Prashad, 1918, *Radiatula* Simpson, 1900, *Scabies* Haas, 1911, *Scabiellus* **gen. nov**., and *Unionetta* Haas, 1955^5,26,27^.

Distribution: This group is widespread throughout the Indian, Western Indochina and Sundaland subregions^5,26,27^.

#### **Genus*Scabiellus*gen. nov**

Figure 2G Type species: *Scabies songkramensis* Kongim & Panha, 2015.

Comments: Monotypic genus (Table 3) representing a distinct phylogenetic lineage that is not sister to the other members of *Scabies*, including its type species, *S. scobinatus* (Lea, 1856) (Fig. 1). Although *Scabiellus* **gen. nov**. is conchologically similar to *Scabies* and several taxa of *Indochinella* by having a v-shaped shell sculpture^30^, this external similarity appears to be only a result of convergence.

Diagnosis: Small mussels, shell length up to 33 mm. Shell thick, rather short, сuneiform, with a broad and elevated umbo, broad anterior margin and narrower posterior margin. Periostracum brown. Dark brown v-shaped sculpture covers the entire shell disc.

Etymology: The name of this genus derived from the genus *Scabies*, in which its type species was described initially.

Distribution: *Scabiellus songkramensis* **gen. & comb. nov**. is recorded from rivers of the Khorat Plateau in Thailand, i.e. the Songkhram and Kam river basins, and the corresponding section of the Mekong River^26,27,30^.

#### Subfamily Gonideinae Ortmann, 1916

Comments: Here we use this subfamily in a broader sense, with the former subfamilies Pseudodontinae^22^ and Rectidentinae^22,23^ being tribes within the monophyletic Gonideinae, as suggested based on the mitogenomic^20^ and multi-locus nuclear^21^ phylogenies.

#### Tribe Contradentini Modell, 1942

Type genus: *Contradens* Haas, 1911 (by original designation)

Comments: This tribe includes six valid genera: *Balwantia* Prashad, 1919, *Contradens* Haas, 1911, *Trapezoideus* Simpson 1900, *Physunio* Simpson, 1900, *Solenaia* Conrad, 1869, and *Yaukthwa* Konopleva *et al*., 2019^7,21^.

Distribution: Northeastern India (Brahmaputra Basin) and Southeast Asia, including the Greater Sunda Islands^2,3,7,29,31^.

#### **Genus*Balwantia*Prashad, 1919 stat. rev**

Type species: *Anodonta soleniformis* Benson, 1836 (by original designation)

Comments: This genus contains ultra-elongated mussels externally resembling members of *Solenaia*^32^ (Fig. 2D) but is distantly related to *Yaukthwa* phylogenetically (Fig. 1). Previously, it was considered a synonym of *Solenaia*^29^ and was not used as a valid genus name since the last monograph of Haas^33^. Two species were recorded from Myanmar, one of which is new to science and described here (Table 3). They were collected from deep burrows which they dig in hard clay and soft sandstone substrate as does *Balwantia soleniformis*, their putative Indian relative from the upper Brahmaputra River^34,35^.

Distribution: Ayeyarwady and upper Brahmaputra basins^29,32,34^.

#### ***Balwantia baniensis*sp. nov**

Figure 2C, 5A, Tables 1–2, Supplementary Table 2

Holotype: RMBH biv0666_2, MYANMAR: Bani River near Bangong village, 19.3247°N, 94.9839°E, Ayeyarwady Basin, 09.xii.2018, Bogan, Bolotov, Vikhrev, Lopes-Lima, Nyein Chan and local villagers leg. Reference sequence numbers of the holotype are as follows: MN275077 (*COI*), MN307264 (*16* *S rRNA*), and MN307206 (*28* *S rRNA*). Shell measurements of the holotype are as follows: SL 57.0 mm, SH 23.2 mm, and SW 15.3 mm.

Paratypes: MYANMAR: type locality, same collecting date, and collectors, 4 specimens (RMBH biv0666_1, biv0666_3, biv0666_4, and biv0666_5), 9 specimens (NCSM 113369).

Etymology: The new species name is dedicated to Bani River, a tributary of the Ayeyarwady River, in which it was collected.

Diagnosis: The new species can be distinguished from *Balwantia elongatula* **comb. nov**. by having a rostrate anterior margin (vs. rounded), a more inflated shell (vs. flattened), and by the presence of bars from umbo along the dorsal margin. The new species also differs from *Balwantia elongatula* **comb. nov**. by fixed nucleotide substitutions in the *COI*, *16* *S rRNA* and *28* *S rRNA* gene fragments (Table 2).

Description: Medium-sized mussel: SL 46.1–61.4 mm, SH 19.5–26.3 mm, SW 12.7–16.7 mm. Shell somewhat trapezoidal, elongated, not very inequilateral, thin, and rather inflated, narrow and rostrate anteriorly, broad and truncated posteriorly, ventral margin slightly curved. Posterior slope covered by elongated, slightly curved bars. Umbo eroded, slightly elevated, without clear sculpture. Periostracum olive-yellow or brownish, the central part of the shell usually lighter than the posterior side. Nacre blue-whitish, sometimes with yellow spots, shining. Lateral teeth very thin, almost straight, by one on each valve. Pseudocardinal teeth reduced. Anterior adductor scars somewhat ovate or drop-like, not deep. Posterior adductor scars ovate or rounded shape, shallow.

Distribution: Bani River, Ayeyarwady Basin, central Myanmar.

#### **Genus*Trapezoideus*Simpson, 1900**

Type species: *Unio foliaceus* Gould, 1843 (by original designation)

Comments: Small genus with a restricted range that was previously considered a monotypic entity^7^. However, we found one additional congeneric species (Table 3), which is described here.

Distribution: Southern Myanmar (Lenya and Dawei basins) and southwestern Thailand (Mae Klong Basin)^7^.

#### ***Trapezoideus lenya*sp. nov**

Figures 2E, 5A, Tables 1–2, Supplementary Table 2

Holotype: RMBH biv0629_2, MYANMAR: 14 Mile Stream, 11.3508°N, 99.1093°E, Lenya River basin, 24.xi.2018, Bogan, Bolotov, Vikhrev, Lopes-Lima, Nyein Chan and local villagers leg. Reference sequence numbers of the holotype are as follows: MN275068 (*COI*), MN307257 (*16* *S rRNA*), and MN307198 (*28* *S rRNA*). Shell measurements of the holotype are as follows: SL 36.3 mm, SH 21.4 mm, and SW 11.3 mm.

Paratypes: MYANMAR: type locality, same collecting date, and collectors, 3 specimens (RMBH biv0629_1, biv0629_3, and biv0629_5), 3 specimens (NCSM 113368).

Etymology: The name of new species is derived from the Lenya River, a coastal freshwater basin in southern Myanmar, from which this species was collected.

Diagnosis: The new species is morphologically similar to *Trapezoideus foliaceus* but differs in shell shape, being higher posteriorly and narrower anteriorly, with a straight ventral margin. The new species also differs from its congener by fixed nucleotide substitutions in the *COI* and *16* *S rRNA* gene fragments (Table 2).

Description: Small mussel: SL 33.1–36.3 mm, SH 19.1–21.4 mm, SW 10.2–11.3 mm. Shell rounded, somewhat trapezoidal, inequilateral, rather thin and compressed, anterior margin rounded and narrow, posterior margin broad and somewhat truncated, dorsal margin high with minute bars extended from the umbo, ventral margin straight. Umbo small, slightly projected, eroded. Periostracum olive-brown. Nacre bluish gray. Pseudocardinal teeth thin and lamellar, two teeth in right valve and one tooth in left valve. Lateral teeth slender, elongated, slightly curved, one in right valve and two in left valve. Anterior adductor scar somewhat drop-like, shallow, posterior adductor scar almost reduced.

Distribution: Lenya River basin, southern Myanmar.

#### **Genus*Yaukthwa*Konopleva*et al****.***, 2019**

Type species: *Trapezoideus nesemanni* Konopleva, Vikhrev & Bolotov, 2017 (by original designation)

Comments: A large genus with at least eight species^7^, including a species newly described here (Table 3).

Distribution: Endemic to the Western Indochina Subregion^7^.

#### ***Yaukthwa avaensis*sp. nov**

Figure 2F, 5A, Tables 1–2, Supplementary Table 2

Holotype: RMBH biv0680_3, MYANMAR: Tarkat Stream, 25.2758°N, 97.2722°E, tributary of the Ayeyarwady River, 23.iii.2018, Nyein Chan leg. Reference sequence numbers of the holotype are as follows: MN275071 (*COI*), MN307259 (*16* *S rRNA*), and MN307200 (*28* *S rRNA*). Shell measurements of the holotype are as follows: SL 38.3 mm, SH 20.8 mm, and SW 14.1 mm.

Paratypes: MYANMAR: type locality, same collecting date, and collector, 9 specimens (RMBH biv0680_1, biv0680_5, biv0680_2, biv0680_4, biv0680_6, biv0680_7, biv0680_8, biv0680_9, and biv0680_10).

Etymology: The name of the new species is derived from the ancient Ava Kingdom in central Myanmar.

Diagnosis: The new species can be distinguished from its sister species *Yaukthwa paiensis* by having a more curved ventral margin and stronger inflation of the shell. The new species also differs from its congeners by fixed nucleotide substitutions in the *COI* gene fragment (Table 2).

Description: Small mussel: SL 24.7–46.6 mm, SH 14.1–25.1 mm, SW 8.7–17.0 mm. Shell subtrapezoidal, inequilateral, moderately thick and rather inflated. Anterior margin rounded, dorsal margin elevated posteriorly, ventral outline slightly curved, posterior slope truncated and usually covered by small striae. Umbo slightly elevated and strongly eroded at some specimens. Periostracum from light to dark brown. Nacre bluish, with yellow spots. Pseudocardinal teeth thin, lamellar, one tooth in the left valve and two teeth in the right valve. Lateral teeth elongated, slightly curved, one in the right valve and two in the left valve. Adductor muscle scars shallow.

Distribution: Middle section of the Ayeyarwady River, central Myanmar.

#### Tribe Pseudodontini Frierson, 1927

Type genus: *Pseudodon* Gould, 1844 (by original designation)

Comments: This tribe includes seven valid genera: *Bineurus* Simpson, 1900, *Monodontina* Conrad, 1853, *Nyeinchanconcha* **gen. nov**., *Pilsbryoconcha* Simpson, 1900, *Sundadontina* **gen. nov**., *Thaiconcha* **gen. nov**. (subtribe Pilsbryoconchina Bolotov, Vikhrev & Tumpeesuwan, 2017), and *Pseudodon* Gould, 1844 (subtribe Pseudodontina s. str.).

Distribution: Southeast Asia from the Ayeyarwady River to the Mekong Basin, Malaysia and the Greater Sunda Islands^2–4,22,31^.

#### Subtribe Pilsbryoconchina Bolotov, Vikhrev & Tumpeesuwan, 2017

Type genus: *Pilsbryoconcha* Simpson, 1900 (by original designation)

#### **Genus*Monodontina*Conrad, 1853**

=*Suborbiculus* Simpson, 1900

Type species: *Margaritana vondembuschiana* Lea, 1840 (by original designation)

Comments: This genus contains seven species (Table 3), three of which are new to science and described here.

Distribution: Sundaland Subregion (Lenya Basin in Myanmar, Mekong Basin, Malaysia, and the Greater Sunda Islands)^4^.

#### ***Monodontina laosica*sp. nov**

Figure 3C, 5A, Tables 1–2, Supplementary Table 2

Holotype: UMMZ 304650, LAOS: ca. 300 m upstream of the mouth of Houai Pin Stream, 14.7944°N, 106.4842°E, a tributary of the Vang Ngao River, Mekong Basin, 21.v.2009, Kottelat *et al*. leg. Reference sequence numbers of the holotype are as follows: KP795029 (*COI*) and KP795052 (*16* *S rRNA*). Shell measurements of the holotype are as follows: SL 61.4 mm, SH 41.4 mm, and SW 19.0 mm.

Etymology: The name of the new species is derived from the country of Laos, in which it was recorded.

Diagnosis: This species can be distinguished from its sister taxa by having a higher dorsal margin and reduced pseudocardinal teeth. The new species also differs from its congeners by fixed nucleotide substitutions in the *COI* and *16* *S rRNA* gene fragments (Table 2).

Description: Medium-sized mussel. Shell ovate, inequilateral, rather inflated, with high dorsal margin, creating a wing, rounded anteriorly, truncated posteriorly, ventral margin curved. Umbo not prominent, eroded. Periostracum brownish with yellow and rusty sites. Nacre blue-whitish with cream tint near the umbo. Pseudocardinal teeth weak. Both muscle scars shallow.

Distribution: Vang Ngao River, Mekong Basin, southern Laos.

#### ***Monodontina lenyanensis*sp. nov**

Figures 3D, 5A, Tables 1–2, Supplementary Table 2

Holotype: RMBH biv0628_2, MYANMAR: 14 Mile Stream, 11.3508°N, 99.1092°E, Lenya River basin, 24.xi.2018, Bogan, Bolotov, Vikhrev, Lopes-Lima, Nyein Chan and local villagers leg. Reference sequence numbers of the holotype are as follows: MN275055 (*COI*), MN307246 (*16* *S rRNA*), and MN307187 (*28* *S rRNA*). Shell measurements of the holotype are as follows: SL 63.4 mm, SH 40.1 mm, and SW 23.7 mm.

Paratypes: MYANMAR: type locality, same collecting date, and collectors, 9 specimens (RMBH biv0628_1, biv0628_3, biv0628_4, biv0628_5, biv0628_6, biv0628_7, biv0628_8, biv0628_9, and biv0628_10), 9 specimens (NCSM 104012).

Etymology: This new species is dedicated to the Lenya River, its type locality.

Diagnosis: This species can be distinguished from its sister taxa by presenting an ovate, elongated, rather solid and inflated shell, not elevated umbo, tubercular or pyramidal pseudocardinal teeth, and rather reduced muscle scars. The new species also differs from its congeners by fixed nucleotide substitutions in the *COI* and *16* *S rRNA* gene fragments (Table 2).

Description: Medium-sized mussel: SL 31.2–63.4 mm, SH 20.3–40.1 mm, SW 11.0–23.7 mm. Shell ovate, elongated, inequilateral, rather solid and inflated, rounded anteriorly, broad and truncated posteriorly, dorsal margin high, ventral margin slightly curved. Umbo small, slightly elevated, eroded. Periostracum rusty-brown, smooth. Nacre white-yellowish. One pseudocardinal tooth in each valve, which is tubercular-like or more pyramidal and sharper, rather high and strong, smooth or slightly ribbed. Lateral teeth reduced. Anterior adductor scar ovate, rather prominent; posterior adductor scar reduced, weakly developed.

Distribution: Lenya River basin, southern Myanmar.

#### ***Monodontina mekongi*sp. nov**

Figures 3E, 5A, Tables 1–2, Supplementary Table 2

Holotype: RMBH biv0122, THAILAND: clay bottom, headwaters of the Phong River, 16.8616°N, 101.9105°E, Mekong Basin, Loei Province, 09.iv.2014, Bolotov, Vikhrev, Spitsyn and Gofarov leg. Reference sequence numbers of the holotype are as follows: KX865861 (*COI*), KX865632 (*16* *S rRNA*), and KX865733 (*28* *S rRNA*). Shell measurements of the holotype are as follows: SL 65.7 mm, SH 42.2 mm, and SW 20.3 mm.

Etymology: The name of this species is derived from the Greater Mekong Basin, its type locality.

Diagnosis: This species is conchologically and genetically related with *Monodontina vondembuschiana* and *M. laosica* **sp. nov.** but it can be distinguished from these species by presenting an uninflated, weak pseudocardinal teeth (vs. stouter), and a curved and lower dorsal margin (vs. strait and higher). The new species also differs from its congeners by fixed nucleotide substitutions in the *COI*, *16* *S rRNA* and *28* *S rRNA* gene fragments (Table 2).

Description: Medium-sized mussel. Shell obovate, slightly higher posteriorly, inequilateral, thin, semitransparent, not inflated; anterior margin rounded, posterior margin angulate, ventral margin curved. Umbo slightly elevated, eroded, without clear sculpture. Periostracum olive yellow. Nacre whitish. Pseudocardinal teeth weak, flatten, more developed in the right valve than in the left one. Both muscle scars shallow, anterior scar irregular; posterior scar somewhat drop-like, almost invisible.

Distribution: Phong River, Mekong Basin, northern Thailand.

#### **Genus*Nyeinchanconcha*gen. nov**

Type species: *Nyeinchanconcha nyeinchani* **gen. & sp. nov**.

Comments: Remarkable monotypic genus (Table 3).

Diagnosis: Shell elliptical, resembling that of the genus *Lamellidens* Simpson, 1900 (Parreysiinae: Lamellidentini), slightly elevated posteriorly, moderately thick and inflated, umbo not elevated, pseudocardinal teeth strong and somewhat pyramidal in each valve; anterior adductor scar drop-like, developed, usually contiguous with pedal retractor scar; the posterior muscle scar shallow.

Etymology: This genus is dedicated to our friend Mr. Nyein Chan, an enthusiastic conservation biologist from FFI – Myanmar Program, Yangon, Myanmar, for his valuable contribution to the conservation of freshwater ecosystems in Southeast Asia. This genus name means “Shell of Nyein Chan” (“concha” being shell in Latin).

Distribution: Mekong Basin in Laos.

#### ***Nyeinchanconcha nyeinchani*gen. & sp. nov**

Figure 3F, 5A, Tables 1–2, Supplementary Table 2

Holotype: NCSM 84884, LAOS: small stream arising at a cave near Ban Kouanphavang, 17.4578°N, 104.9263°E, Nam Done River drainage, Mekong Basin, Khammouane Province, 17.v.2012, M. Kottelat *et al*. leg. Reference sequence numbers of the holotype are as follows: KX822662 (*COI*) and KX822618 (*28* *S rRNA*). Shell measurements of the holotype are as follows: SL 50.9 mm, SH 27.8 mm, and SW 15.6 mm.

Paratypes: LAOS: type locality, same date, and collectors, 1 specimen (NCSM 113351); Nam Phiat River near Phon Bong village, 18.0839°N, 104.9781°E, ca. 2 km from confluence with the Namkading River, Mekong Basin, Bolikhamsai Province, 12.v.2009, 1 specimen (UMMZ 304648), M. Kottelat *et al*. leg.

Etymology: This species is dedicated to our friend Mr. Nyein Chan, a conservation biologist from FFI – Myanmar Program, Yangon, Myanmar.

Diagnosis: The species is morphologically and genetically more similar to *Sundadontina brandti* **sp. nov**. but differs from it by a more elliptical shell without marked elevation of the dorsal margin, and by pyramidal and weaker pseudocardinal teeth. The new species also differs from other Pseudodontini taxa by fixed nucleotide substitutions in the *COI* and *16* *S rRNA* gene fragments (Table 2).

Description: Medium-sized mussel: SL 33.7–59.9 mm, SH 15.1–37.0 mm, SW 7.9–15.6 mm. Shell elliptical, inequilateral, rounded anteriorly, truncated posteriorly, dorsal margin elevated, ventral margin slightly curved. Umbo not prominent, eroded. Periostracum dark brown. Nacre whitish with cream tint near the umbo. Pseudocardinal teeth somewhat pyramidal, stout. Anterior adductor scar pronounced; posterior adductor scar weak.

Distribution: Nam Done and Nam Phiat rivers, Mekong Basin, Laos.

#### **Genus*Sundadontina*gen. nov**

Type species: *Anodonta cumingii* Lea, 1850.

Comments: This genus contains at least 10 species, three of which are new to science and described here (Table 3).

Diagnosis: Shell ovate or elongate-ovate, rather thick and strong, umbo not projected, pseudocardinal teeth stout and tubercular-like; anterior muscle scar ovate and well-developed, usually contiguous with pedal retractor scar; the posterior muscle scar shallow.

Etymology: The name of this genus is derived from that of the genus *Monodontina*, but with another prefix highlighting its broad distribution across the ancient Sundaland.

Distribution: Sundaland Subregion: Lenya Basin in Myanmar, Mekong Basin in Thailand, Cambodia, and southern Vietnam, Chao Phraya Basin in Thailand, Malaysia.

#### ***Sundadontina brandti*sp. nov**

Figures 4B, 5A, Tables 1–2, Supplementary Table 2

Holotype: RMBH biv0475_2, THAILAND: headwater of the Mun River, 14.4138°N, 102.0821°E, Mekong Basin, Khorat Plateau, Nakhon Ratchasima Province, 12.iii.2018, Bolotov, Vikhrev, and local villagers leg. Reference sequence numbers of the holotype are as follows: MN275058 (*COI*), MN307249 (*16* *S rRNA*), and MN307190 (*28* *S rRNA*). Shell measurements of the holotype are as follows: SL 85.4 mm, SH 53.7 mm, and SW 27.7 mm.

Paratypes: THAILAND: type locality, same collecting date, and collectors, 2 specimens (RMBH biv0475_3, biv0475_4).

Etymology: This species is named in the memory of Dr. Rolf Arthur Max Brandt (1917–1989), one of the most influential freshwater malacologists of the last century. This prominent scientist worked in Southeast Asia on freshwater mollusks and authored the freshwater mollusks of Thailand^31^.

Diagnosis: The species is similar to *Sundadontina taskaevi* **sp. nov**. but can be distinguished from it by having a more slender and higher pseudocardinal tooth on the right valve. The new species also differs from its congeners by fixed nucleotide substitutions in the *COI* and *16* *S rRNA* gene fragments (Table 2).

Description: Large mussel: SL 71.3–85.4 mm, SH 43.8–53.7 mm, SW 24.1–27.7 mm. Shell ovate, very inequilateral, solid, not very inflated, rounded anteriorly, truncated posteriorly, dorsal margin curved, ventral margin slightly rounded. Periostracum brownish-black. Nacre creamy. Umbo very small, not developed, eroded. Left valve with one tubercle-like pseudocardinal tooth, right valve with one rectangular and high pseudocardinal tooth. Anterior muscle scar rather well-developed, ovate; posterior muscle scar slightly visible.

Distribution: Mun Basin, Thailand.

#### ***Sundadontina tanintharyiensis*sp. nov**

Figure 4C, 5A, Tables 1–2, Supplementary Table 2

Holotype: RMBH biv0643_4, MYANMAR: Chaung Nauk Pyan Stream, 11.7620°N, 99.1124°E, Lenya River basin, 16.vi.2018, Nyein Chan leg. Reference sequence numbers of the holotype are as follows: MN275057 (*COI*), MN307248 (*16* *S rRNA*), and MN307189 (*28* *S rRNA*). Shell measurements of the holotype are as follows: SL 57.1 mm, SH 40.8 mm, and SW mm.

Paratypes: MYANMAR: type locality, same collecting date, and collectors, 2 specimens (RMBH biv0643_1, biv0643_6) and 3 specimens (NCSM 113364).

Etymology: The new species name is derived from the Tanintharyi Region of Myanmar, in which its type locality is situated.

Diagnosis: The new species is similar to *Sundadontina cumingii* **gen. & comb. nov**. but can be distinguished from it by having a more rounded and inflated shell. The new species also differs from its congeners by fixed nucleotide substitutions in the *COI* and *16* *S rRNA* gene fragments (Table 2).

Description: Medium-sized mussel: SL 39.2–61.2 mm, SH 27.2–45.1 mm, SW 14.7–23.2 mm. Shell ovate or slightly elongated, inequilateral, moderately solid and rather inflated. Anterior margin rounded, dorsal and ventral margin curved, posterior margin subangular. Umbo not elevated, eroded. Periostracum rusty-brown, smooth. Nacre yellowish white. Each valve with one tubercle-like, smooth pseudocardinal tooth. Lateral teeth reduced. Anterior muscle scar ovate or drop-like, rather prominent; posterior muscle scar drop-like and very shallow.

Distribution: Lenya River basin, southern Myanmar.

#### ***Sundadontina taskaevi*sp. nov**

Figures 4D, 5A, Tables 1–2, Supplementary Table 2

Holotype: RMBH biv0475_1, THAILAND: headwater of the Mun River, 14.4138°N, 102.0821°E, Mekong Basin, Khorat Plateau, Nakhon Ratchasima Province, 12.iii.2018, Bolotov and Vikhrev leg. Reference sequence numbers of the holotype are as follows: MN275061 (*COI*), MN307251 (*16* *S rRNA*), and MN307192 (*28* *S rRNA*). Shell measurements of the holotype are as follows: SL 82.7 mm, SH 52.2 mm, and SW 29.3 mm.

Paratypes: THAILAND: type locality, same collecting date, and collectors, 1 specimen (RMBH biv0475_5).

Etymology: This species is named in memory of the late Dr. Anatoly Ivanovich Taskaev (1944–2010), a well-known Russian biologist.

Diagnosis: The new species is similar to *Sundadontina brandti* **sp. nov**. but can be distinguished from it by having broader, stronger, tubercle-like pseudocardinal teeth. The new species also differs from its congeners by fixed nucleotide substitutions in the *COI* and *16* *S rRNA* gene fragments (Table 2).

Description: Large mussel: SL 60.0–82.7 mm, SH 42.8–52.2 mm, SW 22.6–29.3 mm. Shell elongate-ovate, inequilateral, rather solid and inflated, rounded anteriorly, dorsal margin convex, ventral margin slightly curved. Periostracum blackish, with brown lines. Nacre creamy. Umbo slightly elevated, eroded. Each valve with one tubercle-like, smooth pseudocardinal tooth. The tooth in right valve more developed, rather trapezoidal, with broader base. Anterior muscle scar ovate, rather well-developed; posterior muscle scar shallow.

Distribution: Mun River, Thailand.

#### **Genus*Thaiconcha*gen. nov**

Type species: *Anodonta callifera* Martens, 1860.

Comments: This genus contains at least two valid species (Table 3).

Diagnosis: Shell large, thick, elliptical or rounded, moderately inflated. Pseudocardinal teeth rather well developed, muscle attachment scars deep.

Etymology: The name of this genus means “a shell from Thailand”.

Distribution: Mekong Basin in Thailand and Cambodia.

#### ***Subtribe*Pseudodontina Frierson, 1927**

Type genus: *Pseudodon* Gould, 1844 (by original designation)

#### **Genus*Pseudodon*Gould, 1844**

Type species: *Anodon inoscularis* Gould, 1844 (by original designation)

Comments: This genus contains eight species, one of which is new to science and described here (Table 3).

Distribution: Endemic clade to the Western Indochina Subregion^4^.

#### ***Pseudodon kayinensis*sp. nov**

Figure 3G, 5A, Tables 1–2, Supplementary Table 2

Holotype: RMBH biv0618_1, MYANMAR: Winyaw River, 15.6685°N, 97.9496°E, Ataran River basin, 20.xi.2018, Vikhrev, Bogan, Lopes-Lima, and local villagers leg. Reference sequence numbers of the holotype are as follows: MN275043 (*COI*). Shell measurements of the holotype are as follows: SL 59.6 mm, SH 34.9 mm, and SW 17.7 mm.

Paratypes: MYANMAR: type locality, same collecting date, and collectors, 4 specimens (RMBH biv0618_2, biv0618_3, biv0618_4, biv0618_5) and 4 specimens (NCSM 104014); Ko Du Kwe Stream, 15.6132°N, 98.2363°E, Zami River, Ataran River basin, 26.ii.2018, 5 specimens (RMBH biv0637_1, biv0637_2, biv0637_3, biv0637_4, biv0637_5) and 5 specimens (NCSM 113362), Than Win leg.; unnamed stream, 17.0292°N, 97.8100°E, Hlaingbwe River basin, 17.xi.2018, 3 specimens (RMBH biv0638_1, biv0638_2, biv0638_3) and 5 specimens (NCSM 104015), Than Win leg.

Etymology: The name of new species is derived from its distribution range, i.e. the Kayin State in Myanmar.

Diagnosis: The new species is conchologically more similar to *Pseudodon bogani* but can be distinguished from it by having a more curved dorsal margin and clearly ornamented posterior side. The new species also differs from its congeners by fixed nucleotide substitutions in the *COI*, *16* *S rRNA* and *28* *S rRNA* gene fragments (Table 2).

Description: Rather large mussel: SL 37.8–71.0 mm, SH 22.4–42.5 mm, SW 10.3–21.2 mm. Shell from ovate to elliptical, elongated, inequilateral, moderately inflated and thick. Anterior margin rounded, posterior margin somewhat truncated, dorsal margin curved and rather high, ventral margin straight or slightly curved. Umbo not elevated, eroded. Periostracum olive-brown to dark brown, the surface from umbo to posterior margin clearly ribbed, having curved bars covering the entire dorsal margin and then radiate along the posterior slope. Nacre whitish, sometimes with yellow sites. Pseudocardinal teeth high, tubercular-like, in each valve. Anterior adductor scar rather well developed, ovate; posterior adductor scar drop-like, more or less visible.

Distribution: Hlaingbwe and Ataran River basins in southern Myanmar.

## Discussion

### The Isthmus of Kra as a significant biogeographic barrier for the Unionidae

It was shown that freshwater basins of the Malacca Peninsula represent a part of the Sundaland Subregion^16,17^ and that the Dawei and Tanintharyi river basins belong to the Western Indochina Subregion^4,5,7,24^. However, the location of the southern boundary of the Western Indochina Subregion was until now unclear, because freshwater mussel faunas of the southern edge of Myanmar south of the Tanintharyi Basin were almost unknown. It was assumed that the Western Indochinese fauna could spread throughout the western coastal rivers of southern Thailand as far south as the Kangar-Pattani Line (7°N latitude along the Thai-Malay border)^5^ that corresponds to a putative ancient seaway^36,37^. In this study, however, we found that freshwater mussel species inhabiting the Lenya River basin such as *Trapezoideus lenya* **sp. nov**., *Monodontina lenyanensis* **sp. nov**., and *Sundadontina tanintharyiensis* **sp. nov**. belong to the Sundaland fauna, and these species were separated from their sister taxa inhabiting the Mekong River and smaller basins of the Gulf of Thailand drainage during the Miocene, with the last split occurring ca. 6 Myr ago. Based on these results, we can conclude that the boundary between freshwater mussel faunas of the Western Indochina and Sundaland subregions is located along the Tanintharyi – Lenya drainage divide just north of the Isthmus of Kra (Fig. 5B). While freshwater mussel faunas between the Lenya Basin and the Malay Peninsula are poorly known^31^, the fauna of Malaysia contains only typical Sundaland unionid taxa supporting our conclusion^3,16^. There is an admixture of Sundaland's taxa, i.e. *Trapezoideus foliaceus*, in the Dawei River^7,24^ that can reflect an ancient river capture.

This isthmus is a major biogeographic barrier corresponding to a putative ancient seaway^36,37^ that influenced distribution ranges of a plethora of animal and plant taxa^38^ and corresponds to the separation of the Oriental (Indo-Burmese) and Sundaland biotas^39^. A growing body of phylogeographic and phylogenetic research indicates that this barrier is reflected through abrupt changes in bird^40^, frog^41^, snake^42^, lizard^43^, giant centipede^44^ and spider^45^ assemblages around the Isthmus of Kra area. However, such examples are still poorly known among freshwater animals. *Tarebia granifera* (Lamarck, 1816) (Thiaridae), a freshwater snail species, shares two distant species-level lineages probably diverged due to marine transgressions through the Isthmus of Kra approximately 5 Myr ago^46^. The high level of genetic divergence between populations of the giant freshwater prawn *Macrobrachium rosenbergii* (de Man, 1879) (Palaemonidae) clearly supports the existence of a hypothetical seaway north of the Isthmus of Kra^37^. Specimens of the Blue Panchax killifish *Aplocheilus panchax* (Hamilton, 1822) (Aplocheilidae) collected just north of the Isthmus of Kra share clear mtDNA affinities to the Indian clade, while those from localities south of this isthmus represent a separate Sundaic clade^47^. In summary, our novel findings agree with available data on other freshwater and terrestrial taxa revealing the presence of a significant biogeographic barrier at the Isthmus of Kra area. There are two more putative connections via lowlands and river valleys at the Krabi and Songkhla isthmuses (Fig. 5B). Freshwater mussel assemblages of these areas are still unknown, but they undoubtedly belong to the Sundaland fauna.

### Eastern edge of the Indian Subregion

The biogeographic boundary between the Indian and Western Indochina freshwater subregions is located along the Naga Hills, Chin Hills, and Rakhine Yoma ranges separating rivers of the Rakhine Coast from the Ayeyarwady Basin. The eastern edge of the Indian Subregion covers the entire Rakhine Coast of Myanmar with numerous coastal freshwater basins. Freshwater animal species inhabiting this area have clear affinities to the Indian fauna, e.g. the sponge *Corvospongilla ultima* (Annandale, 1910), the polychaete worm *Namalycastis indica* (Southern, 1921), and the bivalves *Novaculina gangetica* Benson, 1830, *Lamellidens marginalis* (Lamarck, 1819)^48,49^, and *Parreysia rakhinensis* **sp. nov**. The freshwater fish fauna of the Rakhine Coast contains numerous local endemic species that are also related to the fauna of the Indian Subcontinent^50–52^, being this area considered a regional hotspot of freshwater fish diversity^53^. Our time-calibrated phylogeny indicates that *Parreysia rakhinensis* **sp. nov**. has close affinities to Indian taxa, and the split between these lineages occurred ca. 5 Myr ago.

### Insights into the genus-level taxonomy of the Southeast Asian Unionidae

Based on our comprehensive multi-locus phylogeny, we introduce four new genera and twelve new species of freshwater mussels from Southeast Asia. *Scabiellus* **gen. nov**. represents a remarkable example of convergent evolution of the shell patterns in freshwater bivalves as it is conchologically similar to members of two other genera of the tribe Indochinellini, i.e. *Scabies* and *Indochinella*. The range of this monotypic genus corresponds to the Khorat Plateau, a putative evolutionary hotspot at the Middle Mekong Basin, that presents high levels of endemism in freshwater animals, e.g. bivalves^26–28^, fish^54^, and softshell turtles^55,56^. In summary, the Indochinellini contains three genera (*Indochinella*, *Indonaia*, and *Radiatula*) west of the Salween – Mekong drainage divide and four genera (*Harmandia*, *Scabies*, *Scabiellus* **gen. nov**., and *Unionetta*) to the east of the same boundary.

The tribe Pseudodontini shares one of the largest monophyletic radiations of freshwater mussels in Southeast Asia, with numerous genus- and species level clades^4,22^ that were traditionally placed into two genera, *Pseudodon* and *Pilsbryoconcha*^29,31^. Based on our multi-locus data set, we had previously resurrected two more valid genera, *Monodontina* and *Bineurus*, and indicated the presence of at least three genus-level clades new to science^4^. To establish an updated taxonomy of the Pseudodontini, in the present study we describe three new genera: *Nyeinchanconcha* **gen. nov**., *Sundadontina* **gen. nov**., and *Thaiconcha* **gen. nov**. The first monotypic genus from the Middle Mekong Basin in central Laos represents another example of shell convergence as it conchologically resembles members of the tribe Lamellidentini by having a rather thin, ovate shell with a strongly reduced hinge plate. *Sundadontina* seems to be a large and conchologically variable genus widespread from the Mekong Basin to Thailand, southern Myanmar, and Malaysia. *Thaiconcha* is a remarkable genus that is phylogenetically sister to *Bineurus* but conchologically differs by having a less elongated, ovate-shaped shell.

### Ancient faunal exchanges between freshwater mussel faunas

The time-calibrated phylogeny suggests that there were several ancient exchanges between faunas of the Sundaland and Western Indochina subregions starting as early as the Late Cretaceous (ca. 80 Myr ago), when the subtribes Pseudodontina and Pilsbryoconchina were separated. The Rectidentini + Contradentini clade most likely evolved within the Sundaland Subregion, with an expansion of a single clade to Western Indochina, while the Indochinellini clade shows an opposite pattern. Both the colonization events were placed in the Late Eocene (ca. 40–46 Myr ago) and were probably triggered by a wet and warm climatic episode during this period^57^. We suggest that these splits may reflect ancient river captures/splits with subsequent colonization/vicariance events in freshwater mussels^22^. The recent geological study suggests that the paleo-Mekong River was established as a large river in the Middle Miocene due to increased erosion during a period of high monsoon precipitation^58^. Other research assumes that the paleo-Ayeyarwady probably originated sometime between the Late Eocene and Early Oligocene^59^. However, our results indicate that these ages might be underestimated, and the paleo-Mekong and paleo-Ayeyarwady rivers could have been initiated since the Late Cretaceous as did the paleo-Yangtze System^60^. Two ancient monophyletic mussel radiations (age 51–55 Myr) were previously discovered within the putative paleo-Mekong catchment^22^ also suggesting at least the Early Eocene age of this freshwater system. In summary, our findings support the hypothesis that Southeast Asian freshwater bivalve fauna primarily originated within three evolutionary hotspots (Western Indochina, Sundaland, and East Asian)^5,6^ supplemented by ancient (Late Miocene) immigrants that colonized freshwater systems of the western coast of Myanmar from the Indian Subcontinent.

## Methods

### Data sampling

Mussel specimens were collected from various water bodies throughout Myanmar, Thailand and northern Laos from 2012 to 2018. A foot tissue snip from each specimen was preserved in 96% ethanol immediately after collection. To find the boundaries between biogeographic subregions, we collected freshwater mussels throughout small and medium-sized freshwater basins of the Rakhine Coast and the southern edge of Myanmar in 2018 under a National Geographic Society grant No. NGS-274R-18.

### Studied museum collections

The freshwater mussel shell lots were studied in the malacological collections of the Russian Museum of Biodiversity Hotspots [**RMBH**], Federal Center for Integrated Arctic Research, Russian Academy of Sciences, Arkhangelsk, Russia, National Museum of Natural History [**NMNH**], Smithsonian Institution, Washington, DC, United States of America, British Museum of Natural History [**NHMUK**], London, United Kingdom, Muséum National d’Histoire Naturelle [**MNHN**], Paris, France, Museo Civico di Storia Naturale di Genova [**MSNG**], Genoa, Italy, California Academy of Natural Sciences, San Francisco, United States of America [**CAS**], North Carolina Museum of Natural Sciences [**NCSM**], Raleigh, United States of America, and the University of Michigan Museum of Zoology [**UMMZ**], Ann Arbor, United States of America.

### Molecular data and phylogenetic analyses

Multi-locus phylogeny (3 codons of *COI* + *16* *S rRNA* *+* *28* *S rRNA*) was reconstructed using 271 haplotypes of the Parreysiinae and Gonideinae members from Southeast Asia, East Asia, India, and Africa (Supplementary Table 1). Representatives of the Margaritiferidae, Iridinidae, Etheriidae, Mycetopodidae, Hyriidae, and Trigoniidae were used as outgroup. We used IQ-TREE v1.6.11^61^ and MrBayes v3.2.6^62^ as described in our previous work^6^. Bayesian calculations were performed at the San Diego Supercomputer Center through the CIPRES Science Gateway^63^. The best-fit evolutionary models applied to each partition in the IQ-TREE and MrBayes runs based on Bayesian Information Criterion (BIC) of Model Finder implemented in the IQ-TREE web server^61^ were as follows: F81 + G (1st codon of *COI*); GTR + G (2nd codon of *COI*); TN + I + G (3rd codon of *COI*); GTR + I + G (*16* *S rRNA*); and TIM2 + I + G (*28* *S rRNA*).

### Time-calibrated phylogeny

The time-calibrated phylogeny was reconstructed in BEAST v2.6.1^64^ based on an external *COI* evolutionary rate (0.265 ± 0.06% substitutions per site per million years) estimated for the Unionidae^65^. This rate can be considered a reliable estimate as it is largely congruent with the data inferred from a mitogenomic reconstruction^20^. The same multi-locus dataset as for the IQ-TREE and MrBayes phylogenetic analyses (3 codons of *COI* + *16* *S rRNA* *+* *28* *S rRNA*) was estimated. The evolutionary rate was implemented only to the *COI* partition. The HKY + G model was applied for each gene partition. The analyses were run using a lognormal relaxed clock algorithm with the Yule speciation process as the tree priors^66,67^. Calculations were performed at the San Diego Supercomputer Center through the CIPRES Science Gateway^63^. We conducted four searches, each with 5 × 10^7^ generations and tree sampling every 1000th generation. The log files were checked visually with Tracer v. 1.7^68^. Most of ESS values were recorded as > 300, a few of them were registered > 100. All runs were compiled with LogCombiner v1.8.4^67^ using an additional re-sampling every 10,000th generation and 25% burn-in. The maximum clade credibility tree was obtained using TreeAnnotator v1.8.4^67^.

### Statistical biogeographic analyses

To reconstruct ancestral areas with RASP v3.2^69^, we used the set of 15,004 time-calibrated binary trees that were combined from the four runs of BEAST v2.6.1 (see above). As a condensed tree, we used the user-specified consensus tree, which was calculated based on this set of trees with TreeAnnotator v1.8.4 (see above). Non-target sequences (outgroup taxa and species, the ranges of which are situated beyond Southeast Asia and the Indian Subcontinent) were removed from the tree set using the appropriate option of the software. We used only one haplotype per species. Ancestral area patterns were reconstructed using two probabilistic algorithms: Statistical Dispersal-Vicariance Analysis (S-DIVA) and Bayesian MCMC analysis. Three possible distribution areas were assigned as follows: (A) Western Indochina, (B) Sundaland, and (C) Indian subregions. The S-DIVA analyses were calculated with the following parameters: max areas = 3; allow reconstruction with max reconstructions = 100; max reconstructions for final tree = 1,000; and allowing extinctions. The MCMC analysis was performed with default settings and 500,000 generations. In addition to the reconstructions obtained from each analysis separately, we used summary results of the two kinds of analyses, which were combined with RASP v3.2^69^.

### Species delimitation and diagnostics of new taxa

To delimit and diagnose species in our dataset, we used an integrative approach^4–7,70–73^ based on the phylogenetic and morphological analyses. First, we applied an automatic species delimitation approach to delimit the Molecular Operational Taxonomic Units (MOTUs) that may correspond to biological species. The maximum likelihood *COI* phylogeny of each tribe inferred from IQ-TREE v1.6.11^61^ was used as an input tree for the Poisson Tree Process (PTP) modeling through the PTP web-service (http://mptp.h-its.org)^74^. An uncorrected *COI* mean p-distance to the nearest neighbor of each species-level lineage was calculated in MEGA7^75^. Second, each MOTU within the clades of interest was studied using morphological criteria (shell shape, umbo position, structure of pseudocardinal and lateral teeth, shape of muscle attachment scars), and was compared with the original descriptions of nominal taxa to link each clade to a biological species. Three shell dimensions of each specimen, included in the type series of new taxa, i.e., the length, height, and width of the shell (all at the maximum diameter), were measured using calipers ( ± 0.1 mm) (Table 1 and Supplementary Table 2). The molecular diagnosis of every new species was designed using fixed nucleotide substitutions, which were estimated for each gene separately using a Toggle Conserved Sites tool of MEGA7^75^ at a 50% level. For the diagnoses, an alignment of congeneric haplotype sequences (tribe-level alignment for *Nyeinchanconcha nyeinchani* **gen. & sp. nov**.) was performed using the Muscle algorithm implemented in MEGA7^75^. All deleterious mutations were retained for the analyses. While numerous recent studies reveal that using an integrative approach for freshwater mussel taxonomic research is rather straightforward^4–7,70–73^, its application to freshwater gastropods is more difficult due to several shortcomings such as a possible incongruence in a mitochondrial phylogeny^76,77^ and often higher DNA barcoding thresholds between species^46,78,79^. At first glance, the differences between these groups can be explained by slower evolutionary rates of freshwater mussels^20,65^ compared with those of freshwater gastropods^80^.

### Nomenclatural acts

The electronic edition of this article conforms to the requirements of the amended International Code of Zoological Nomenclature (ICZN), and hence the new names contained herein are available under that Code from the electronic edition of this article. This published work and the nomenclatural acts it contains have been registered in ZooBank (http://zoobank.org), the online registration system for the ICZN. The LSID for this publication is: urn:lsid:zoobank.org:pub:C6AF4F5B-8526–4FF6-BF08-D6697BE24E66. The electronic edition of this paper was published in a journal with an ISSN and has been archived and is available from PubMed Central.

## Supplementary information

Supplementary Information.

## Data Availability

The type series of the new taxa are deposited in the Russian Museum of Biodiversity Hotspots [**RMBH**], Federal Center for Integrated Arctic Research, Russian Academy of Sciences, Arkhangelsk, Russia; the North Carolina Museum of Natural Sciences [**NCSM**], Raleigh, United States of America; and the University of Michigan Museum of Zoology [**UMMZ**], Ann Arbor, United States of America. The molecular sequences obtained in this study are available in GenBank. Sequence accession numbers and collecting locality for each specimen are presented in Supplementary Tables 1–2. Shell measurements for the type series of the new species are given in Table 1 and Supplementary Table 2.
