# Supplementary material for: New freshwater mussel taxa discoveries clarify biogeographic division of Southeast Asia
Source: Sci Rep. 2020 Apr 20;10:6616. doi: 10.1038/s41598-020-63612-5 (PMC7171101; doi:10.1038/s41598-020-63612-5)
Supplement: Supplementary file 1 — Supplementary Information. [file 41598_2020_63612_MOESM1_ESM.pdf]

SUPPLEMENTARY INFORMATION

New freshwater mussel taxa discoveries clarify biogeographic division of Southeast Asia

Ivan N. Bolotov, Ekaterina S. Konopleva, Ilya V. Vikhrev, Mikhail Yu. Gofarov, Manuel Lopes-Lima, Arthur E. Bogan, Zau Lunn, Nyein Chan, Than Win, Olga V. Aksenova, Alena A. Tomilova, Kitti Tanmuangpak, Sakboworn Tumpeesuwan & Alexander V. Kondakov

*Corresponding author: inepras@yandex.ru

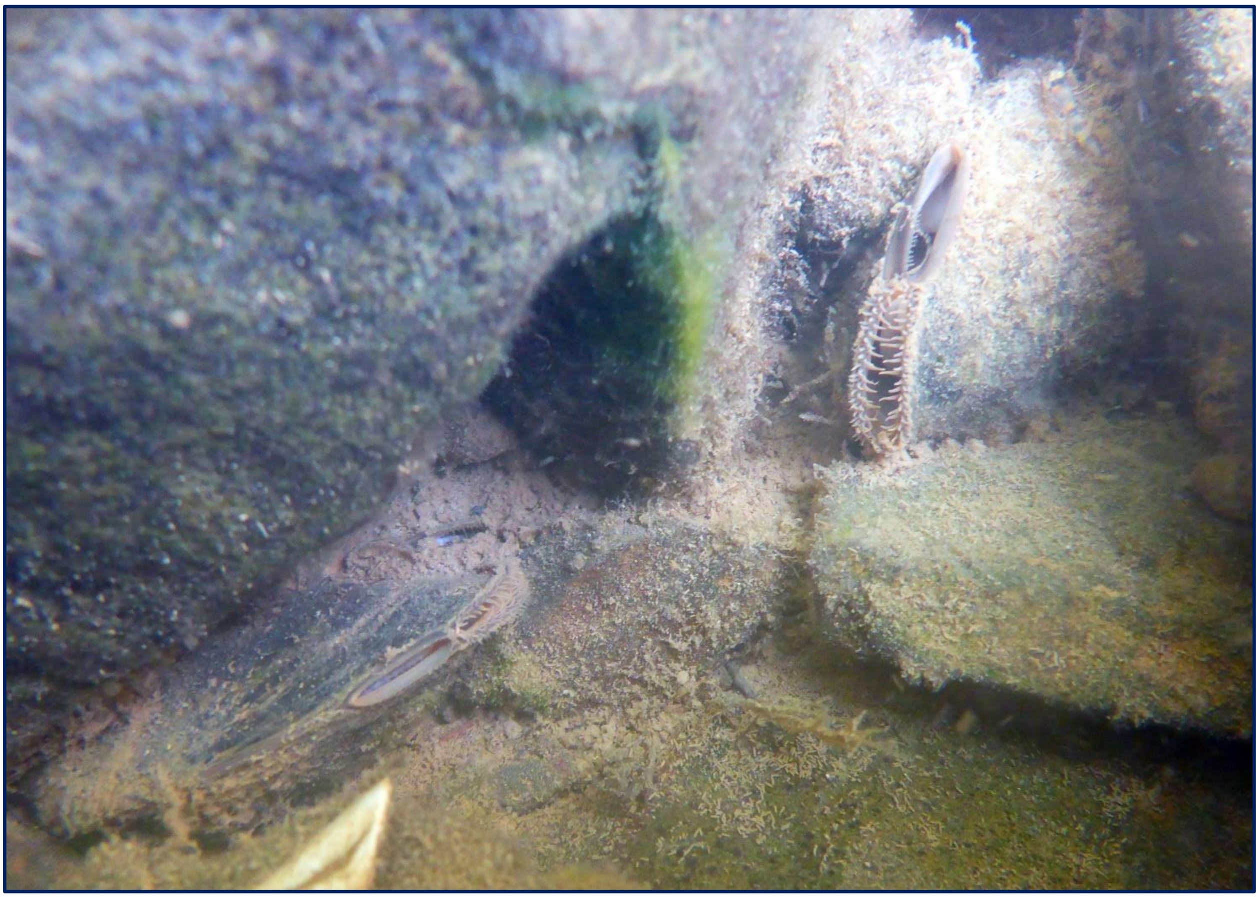

Two live individuals of *Parreysia rakhinensis* **sp. nov.** in the Kyeintali Chaung, Rakhine State, western Myanmar (Photo: Nyein Chan)

Contents

Supplementary Tables

Supplementary Table 1. List of sequences used in this study

Supplementary Table 2. Shell measurements and reference DNA sequences for the type series of new freshwater mussel species (Unionidae) from Southeast Asia

Supplementary Table 3. The most probable ancestral areas of the primary clades within freshwater mussels from Southeast Asia and India inferred from three different statistical modeling approaches. High support values (probability ≥70 %) are highlighted in bold

Supplementary Figures

Supplementary Figure 1. Maximum likelihood phylogeny of the Parreysiinae and Gonideinae based on the complete data set of mitochondrial and nuclear sequences (five partitions: three codons of COI + 16S rRNA + 28S rRNA).

Supplementary Table 1. List of sequences used in this study

Species	Locality	Specimen Voucher	NCBI's GenBank acc. nos.		
			COI	*16S rRNA*	*28S rRNA*
**In-group taxa**					
**UNIONIDAE Rafinesque, 1820**					
**GONIDEINAE Ortmann, 1916**					
**PSEUDODONTINI Frierson, 1927**					
**PSEUDODONTINA s. str.**					
Pseudodon cf. *inoscularis* (Gould, 1844)	Myanmar: Ayeyarwady River basin, a tributary of Lake Indawgyi	RMBH biv0110_5	KX865858	KX865859	KX865860
Pseudodon cf. *inoscularis* (Gould, 1844)	Myanmar: Ayeyarwady River basin, a tributary of Lake Indawgyi	RMBH biv0110_10	KX865629	KX865630	KX865631
Pseudodon cf. *inoscularis* (Gould, 1844)	Myanmar: Ayeyarwady River basin, a tributary of Lake Indawgyi	RMBH biv0110_11	KX865730	KX865731	KX865732
Pseudodon bogani Bolotov, Kondakov & Konopleva, 2017	Myanmar: Sittaung River basin, Kanni River	RMBH biv0241_4	MF352216	MF352290	MF352348
Pseudodon bogani Bolotov, Kondakov & Konopleva, 2017	Myanmar: Sittaung River basin, Kanni River	RMBH biv0241_5	MF352217	MF352291	MF352349
Pseudodon bogani Bolotov, Kondakov & Konopleva, 2017	Myanmar: Sittaung River basin, Kanni River	RMBH biv0241_8	MF352218	MF352292	MF352350
Pseudodon manueli Konopleva, Kondakov & Vikhrev, 2017	Myanmar: Sittaung River basin, Pyowne River	RMBH biv0246_1	MF352228	MF352300	MF352358
Pseudodon manueli Konopleva, Kondakov & Vikhrev, 2017	Myanmar: Sittaung River basin, Pyowne River	RMBH biv0246_3	MF352229	MF352301	MF352359
Pseudodon manueli Konopleva, Kondakov & Vikhrev, 2017	Myanmar: Sittaung River basin, Pyowne River	RMBH biv0246_8	MF352230	MF352302	MF352360
Pseudodon salwenianus (Gould, 1844)	Myanmar: Salween River basin, unnamed stream	RMBH biv0639_1	MN275037	MN307237	MN307178
Pseudodon salwenianus (Gould, 1844)	Myanmar: Salween River basin, unnamed stream	RMBH biv0639_2	MN275038	n/a	n/a
Pseudodon salwenianus (Gould, 1844)	Myanmar: Salween River basin, unnamed stream	RMBH biv0639_3	MN275039	n/a	n/a
Pseudodon salwenianus (Gould, 1844)	Myanmar: Salween River basin, Hlaingbwe Stream	RMBH biv0674_1	MN275040	MN307238	MN307179
Pseudodon salwenianus (Gould, 1844)	Myanmar: Salween River basin, Hlaingbwe Stream	RMBH biv0674_2	MN275041	MN307239	MN307180
Pseudodon salwenianus (Gould, 1844)	Myanmar: Salween River basin, Hlaingbwe Stream	RMBH biv0674_3	MN275042	n/a	n/a

Species	Locality	Specimen Voucher	NCBI's GenBank acc. nos.		
			COI	*16S rRNA*	*28S rRNA*
Pseudodon kayinensis **sp. nov.**	Myanmar: Ataran River basin, Winyaw River	RMBH biv0618_1	MN275043	n/a	n/a
Pseudodon kayinensis **sp. nov.**	Myanmar: Ataran River basin, Winyaw River	RMBH biv0618_2	MN275044	n/a	n/a
Pseudodon kayinensis **sp. nov.**	Myanmar: Ataran River basin, Winyaw River	RMBH biv0618_3	MN275045	MN307240	MN307181
Pseudodon kayinensis **sp. nov.**	Myanmar: Ataran River basin, Zami River, Ko Du Kwe Stream	RMBH biv0637_1	MN275046	MN307241	MN307182
Pseudodon kayinensis **sp. nov.**	Myanmar: Ataran River basin, Zami River, Ko Du Kwe Stream	RMBH biv0637_2	MN275047	n/a	n/a
Pseudodon kayinensis **sp. nov.**	Myanmar: Ataran River basin, Zami River, Ko Du Kwe Stream	RMBH biv0637_3	MN275048	n/a	n/a
Pseudodon kayinensis **sp. nov.**	Myanmar: Hlaingbwe River basin, unnamed stream	RMBH biv0638_1	MN275049	MN307242	MN307183
Pseudodon kayinensis **sp. nov.**	Myanmar: Hlaingbwe River basin, unnamed stream	RMBH biv0638_2	MN275050	n/a	n/a
Pseudodon kayinensis **sp. nov.**	Myanmar: Hlaingbwe River basin, unnamed stream	RMBH biv0638_3	MN275051	n/a	n/a
**PILSBRYOCONCHINA Bolotov, Vikhrev & Tumpeesuwan, 2017**					
Pilsbryoconcha compressa (Martens, 1860)	Thailand: Mekong River basin, Huai Nam Khu Reservoir	RMBH biv0118	KX865875	KX865646	KX865746
Pilsbryoconcha compressa (Martens, 1860)	Thailand: Mekong River basin, artificial pond near the Ban Nong-Bua village	RMBH biv0116_1	KX865872	KX865643	KX865744
Pilsbryoconcha compressa (Martens, 1860)	Thailand: Mekong River basin, artificial pond near the Ban Nong-Bua village	RMBH biv0116_2	KX865873	KX865644	KX865745
Pilsbryoconcha lemeslei (Morelet, 1875)	Vietnam	n/a	KX822657	n/a	n/a
Pilsbryoconcha exilis (Lea, 1838)	Malaysia	X213	KX051289	n/a	n/a
Bineurus mouhotii (Lea, 1863)	Laos: Mekong River basin, Nam Long River	RMBH biv0182_2	KX865876	KX865647	KX865747
Bineurus mouhotii (Lea, 1863)	Laos: Mekong River basin, Nam Long River	RMBH biv0182_19	KX865878	KX865649	KX865749
Bineurus mouhotii (Lea, 1863)	Laos: Mekong River basin, a tributary of Nam Fa River near Vieng Phou Kha	RMBH biv0201_2	KY561623	KY561641	KY561655
Bineurus sp.	Thailand: Mekong River basin, Loei River	RMBH biv0119_1	KX865879	KX865650	KX865750
Bineurus sp.	Thailand: Mekong River basin, Loei River	RMBH biv0119_2	KX865880	KX865651	KX865751
Bineurus sp.	Thailand: Mekong River basin, Loei River	RMBH biv0119_3	KX865881	KX865652	KX865752

Species	Locality	Specimen Voucher	NCBI's GenBank acc. nos.		
			COI	***16S rRNA***	***28S rRNA***
Bineurus exillis (Morelet, 1866)	Thailand: Mekong River basin, Mun River, up-stream of upper reservoir	RMBH biv0474_1	MN275052	MN307243	MN307184
Bineurus exillis (Morelet, 1866)	Thailand: Mekong River basin, Mun River, up-stream of upper reservoir	RMBH biv0474_3	MN275053	MN307244	MN307185
Bineurus exillis (Morelet, 1866)	Laos: Mekong River basin	MMZ 304649	KP795026	KP795051	KP795009
*Monodontina vondembuschiana (*Lea, 1840)	Malaysia	BIV1721	KX051296	n/a	n/a
*Monodontina vondembuschiana (*Lea, 1840)	Malaysia	BIV1806	KX051303	n/a	n/a
*Monodontina vondembuschiana (*Lea, 1840)	Malaysia	X157	KX051306	n/a	n/a
Monodontina mekongii **sp. nov.**	Thailand: Mekong River basin, Phong River	RMBH biv0122	KX865861	KX865632	KX865733
Monodontina laosica **sp. nov.**	Laos: Mekong River basin, tributary of the Vang Ngao River	UMMZ 304650	KP795029	KP795052	n/a
Monodontina cambodiensis (Petit de la Saussaye, 1865)	Cambodia: Mekong River basin, Tonle Sap River: Pursat River	UMMZ 304350	KP795028	KF011262	KP795011
Monodontina cambodiensis (Petit de la Saussaye, 1865)	Malaysia	X198	KX051297	n/a	n/a
Monodontina cambodiensis (Petit de la Saussaye, 1865)	Thailand	n/a	KX822660	n/a	KX822616
Monodontina lenyanensis **sp. nov.**	Myanmar: Lenya River basin, 14 Mile Stream	RMBH biv0628_1	MN275054	MN307245	MN307186
Monodontina lenyanensis **sp. nov.**	Myanmar: Lenya River basin, 14 Mile Stream	RMBH biv0628_2	MN275055	MN307246	MN307187
Monodontina lenyanensis **sp. nov.**	Myanmar: Lenya River basin, 14 Mile Stream	RMBH biv0628_3	MN275056	MN307247	MN307188
Sundadontina cumingii (Lea, 1850) **gen. & comb. nov.**	Malaysia	X115	KX051295	n/a	n/a
Sundadontina cumingii (Lea, 1850) **gen. & comb. nov.**	Malaysia	X79	KX051292	n/a	n/a
Sundadontina tumida (Morelet, 1866) **comb. nov.**	Cambodia: Mekong River basin	UMMZ 304349	KP795027	KF011261	KP795010
Sundadontina tanintharyiensis **sp. nov.**	Myanmar: Lenya River basin, Chaung Nauk Pyan Stream	RMBH biv0643_4	MN275057	MN307248	MN307189
Sundadontina brandti **sp. nov.**	Thailand: Mekong River basin, Mun River	RMBH biv0475_2	MN275058	MN307249	MN307190
Sundadontina brandti **sp. nov.**	Thailand: Mekong River basin, Mun River	RMBH biv0475_3	MN275059	MN307250	MN307191
Sundadontina brandti **sp.**	Thailand: Mekong River	RMBH biv0475_4	MN275060	n/a	n/a

Species	Locality	Specimen Voucher	NCBI's GenBank acc. nos.		
			COI	***16S rRNA***	***28S rRNA***
**nov.**	basin, Mun River				
Sundadontina taskaevi **sp. nov.**	Thailand: Mekong River basin, Mun River	RMBH biv0475_1	MN275061	MN307251	MN307192
Sundadontina taskaevi **sp. nov.**	Thailand: Mekong River basin, Mun River	RMBH biv0475_5	MN275062	n/a	n/a
Thaiconcha callifera (Martens, 1860) **gen. & comb. nov.**	Thailand: Mekong River basin, Phong River	RMBH biv0120_3	KX865865	KX865636	KX865737
Thaiconcha callifera (Martens, 1860) **gen. & comb. nov.**	Thailand: Mekong River basin, Phong River	RMBH biv0120_8	KX865866	KX865637	KX865738
Thaiconcha callifera (Martens, 1860) **gen. & comb. nov.**	Thailand: Mekong River basin, Phong River	RMBH biv0120_11	KX865869	KX865640	KX865741
Thaiconcha sp.	Thailand: Mekong River basin, Mun River	RMBH biv0462	MN275063	MN307252	MN307193
Thaiconcha sp.	Thailand: Mekong River basin, Mun River	RMBH biv0468_1	MN275064	MN307253	MN307194
Thaiconcha sp.	Thailand: Mekong River basin, Mun River	RMBH biv0468_2	MN275065	MN307254	MN307195
Thaiconcha sp.	Thailand: Mekong River basin, Mun River	RMBH biv0468_3	MN275066	MN307255	MN307196
Nyeinchanconcha nyeinchani **gen. & sp. nov.**	Laos: Mekong River basin, Nam Phiat River	UMMZ 304648	KP795025	KP795050	KP795008
Nyeinchanconcha nyeinchani **gen. & sp. nov.**	Laos: Mekong River basin	NCSM 84884	KX822662	n/a	KX822618
**CONTRADENTINI Modell, 1942**					
Contradens contradens (Lea, 1838)	West Malaysia: Pahang River	ANSP 389059	DQ191411	n/a	AF400692
Contradens contradens (Lea, 1838)	Malaysia	RMBH biv0211_2	MF352289	n/a	MF352406
Contradens contradens (Lea, 1838)	Malaysia	BIV1777	KX051262	n/a	n/a
Contradens aff. *contradens* (Lea, 1838) sp.1	Cambodia: Mekong River basin	UMMZ 304652	KP795034	KP795054	KP795016
Contradens eximius (Lea, 1856)	Thailand: Mekong River basin, Chi River	RMBH biv0127 _3	KX865938	KX865686	KX865809
Contradens eximius (Lea, 1856)	Thailand: Mekong River basin, Phong River	RMBH biv0121 _1	KX865939	KX865687	KX865810
Contradens eximius (Lea, 1856)	Thailand: Mekong River basin, Phong River	RMBH biv0121_2	KX865940	KX865688	KX865811
Contradens comptus (Deshayes & Jullien, 1874)	Thailand: Mekong River basin, Loei River	RMBH biv0119_5	KX865928	KX865682	KX865799
Contradens comptus (Deshayes & Jullien, 1874)	Laos: Mekong River basin, Nam Long River	RMBH biv0182_3	KX865930	KX865684	KX865801
Contradens comptus (Deshayes & Jullien, 1874)	Laos: Mekong River basin, Nam Pe River	RMBH biv0185_2	KX865935	n/a	KX865806

Species	Locality	Specimen Voucher	NCBI's GenBank acc. nos.		
			COI	***16S rRNA***	***28S rRNA***
Contradens misellus (Morelet, 1865)	Thailand: Chao Phraya River basin, Ping River	2014-0685	MH345986	MH346026	MH346006
Contradens misellus (Morelet, 1865)	Thailand: Chao Phraya River basin, Ping River	2014-0687	MH345987	MH346027	MH346007
Contradens pallegoixi (Sowerby, 1867)	Thailand: Mekong River Basin, Mun River drainage, Pao River	2014-0613	MH345988	MH346028	MH346008
Contradens pallegoixi (Sowerby, 1867)	Thailand: Mekong River Basin, Mun River drainage, Pao River	2014-0614	MH345989	MH346029	MH346009
Contradens pallegoixi (Sowerby, 1867)	Thailand: Mekong River Basin, Mun River drainage, Pao River	2014-0616	MH345990	MH346030	MH346010
Contradens rolfbrandti Jeratthitikul & Panha, 2019	Thailand: Huai Luang River at Don Kloi Village, Phibun Rak District, Udon Thani Province	UNI0213	MG582016	n/a	n/a
Contradens rolfbrandti Jeratthitikul & Panha, 2020	Thailand: Huai Luang River at Don Kloi Village, Phibun Rak District, Udon Thani Province	UNI0234	MG582017	n/a	n/a
Contradens rolfbrandti Jeratthitikul & Panha, 2021	Thailand: Huai Luang River at Don Kloi Village, Phibun Rak District, Udon Thani Province	UNI0229	MG582018	n/a	n/a
Contradens crossei Deshayes, 1874	Laos: Middle Mekong basin	UNI1174	MG582013	n/a	n/a
Contradens crossei Deshayes, 1875	Laos: Middle Mekong basin	UNI0956	MG582012	n/a	n/a
Contradens crossei Deshayes, 1876	Laos: Middle Mekong basin	UNI0271	MG582008	n/a	n/a
Contradens novoselovi Konopleva et al., 2019	Laos: Mekong River basin, a tributary of Nam Fa River near Vieng Phou Kha	RMBH biv0202_2	KY561630	KY561645	KY561662
Contradens novoselovi Konopleva et al., 2019	Laos: Mekong River basin, a tributary of Nam Fa River near Vieng Phou Kha	RMBH biv0203_5	KY561631	KY561646	KY561663
Contradens novoselovi Konopleva et al., 2019	Laos: Mekong River basin, a tributary of Nam Fa River near Vieng Phou Kha	RMBH biv0203_4	KY561632	KY561647	KY561664
Trapezoideus foliaceus (Gould, 1843)	Thailand: Mae Klong river basin, Pracham Mai River	2012-0443	MH345979	MH346019	MH345999
Trapezoideus foliaceus (Gould, 1843)	Thailand: Mae Klong River basin, Pachee River	ICH-02104	MH345984	MH346024	MH346004
Trapezoideus foliaceus (Gould, 1843)	Thailand: Mae Klong River basin, Pachee River	ICH-02105	MH345985	MH346025	MH346005
Trapezoideus lenya **sp. nov.**	Myanmar: Lenya River basin, 14 Mile Stream	RMBH biv0629_1	MN275067	MN307256	MN307197
Trapezoideus lenya **sp. nov.**	Myanmar: Lenya River basin, 14 Mile Stream	RMBH biv0629_2	MN275068	MN307257	MN307198
Trapezoideus lenya **sp.**	Myanmar: Lenya River	RMBH biv0629_3	MN275069	MN307258	MN307199

Species	Locality	Specimen Voucher	NCBI's GenBank acc. nos.		
			COI	*16S rRNA*	*28S rRNA*
**nov.**	basin, 14 Mile Stream				
Physunio modelli Brandt, 1974	Thailand: Mekong River basin, Chi River	RMBH biv0125_2	KX865883	KX865654	KX865754
Physunio modelli Brandt, 1974	Thailand: Mekong River basin, Chi River	RMBH biv0125_3	KX865884	KX865655	KX865755
Physunio modelli Brandt, 1974	Thailand: Mekong River basin, Chi River	RMBH biv0131	KX865888	n/a	KX865759
Physunio sp.'Contradens'	Cambodia: Mekong River basin	UMMZ 304653	KP795035	KP795055	KP795017
Physunio sp.'Trapezoideus'	Laos: Mekong River basin, Nam Ou River	UMMZ 304347/ UMMZ MC 304347	KP795036	KF011265	KP795018
Physunio superbus (Lea, 1843)	Malaysia	X180	KX051282	n/a	n/a
Physunio superbus (Lea, 1843)	Malaysia	BIV1776	KX051278	n/a	n/a
Physunio superbus (Lea, 1843)	Malaysia	X246	KX051275	n/a	n/a
Yaukthwa nesemanni (Konopleva, Vikhrev & Bolotov, 2017)	Myanmar: Sittaung River basin, Thauk Ye Kupt River	RMBH biv0144_14	KX865906	KX865663	KX865777
Yaukthwa nesemanni (Konopleva, Vikhrev & Bolotov, 2017)	Myanmar: Sittaung River basin, Thauk Ye Kupt River	RMBH biv0144_19	KX865908	KX865665	KX865779
Yaukthwa nesemanni (Konopleva, Vikhrev & Bolotov, 2017)	Myanmar: Sittaung River basin, Thauk Ye Kupt River	RMBH biv0255_2	MF352254	n/a	MF352379
Yaukthwa panhai (Konopleva, Bolotov & Kondakov, 2017.)	Myanmar: Sittaung River basin, Kyan Hone River	RMBH biv0138_4	KX865909	KX865666	KX865780
Yaukthwa panhai (Konopleva, Bolotov & Kondakov, 2017.)	Myanmar: Sittaung River basin, Kyan Hone River	RMBH biv0138_12	KX865913	KX865670	KX865784
Yaukthwa panhai (Konopleva, Bolotov & Kondakov, 2017.)	Myanmar: Sittaung River basin, Kyan Hone River	RMBH biv0155_4	KX865911	KX865668	KX865782
Yaukthwa inlenensis Konopleva et al., 2019	Myanmar: Salween River basin, Inle Lake Channel	RMBH biv0114_1	KX865915	KX865672	KX865786
Yaukthwa inlenensis Konopleva et al., 2019	Myanmar: Salween River basin, Mway Stream	RMBH biv0139_7	KX865922	KX865676	KX865793
Yaukthwa inlenensis Konopleva et al., 2019	Myanmar: Salween River basin, Nam Pilu River	RMBH biv0140_24	KX865926	KX865680	KX865797
Yaukthwa paiensis Konopleva et al., 2019	Thailand: Salween River basin, Khong River	ICH-00638	MH345970	MH346011	MH345991
Yaukthwa paiensis Konopleva et al., 2019	Thailand: Salween River basin, Khong River	ICH-00639	MH345971	MH346012	MH345992
Yaukthwa paiensis Konopleva et al., 2019	Thailand: Salween River basin, Khong River	ICH-00640	MH345972	n/a	n/a
Yaukthwa avaensis **sp. nov.**	Myanmar: Ayerrawaddy River basin, Tarkat Stream	RMBH biv0680_1	MN275070	n/a	n/a
Yaukthwa avaensis **sp. nov.**	Myanmar: Ayerrawaddy River	RMBH biv0680_3	MN275071	MN307259	MN307200

Species	Locality	Specimen Voucher	NCBI's GenBank acc. nos.		
			COI	*16S rRNA*	*28S rRNA*
	basin, Tarkat Stream				
Yaukthwa avaensis **sp. nov.**	Myanmar: Ayerrawaddy River basin, Tarkat Stream	RMBH biv0680_5	MN275072	n/a	MN307201
Yaukthwa cf. *dalliana* (Frierson, 1913)	Myanmar: Ayeyarwady River basin, Nanyinhka Chaung River	RMBH biv011_2	KX865889	KX865656	KX865760
Yaukthwa cf. *dalliana* (Frierson, 1913)	Myanmar: Ayeyarwady River basin, Mali Hka River basin, Pan Khai Stream	RMBH biv0101_5	KX865894	KX865659	KX865763
Yaukthwa cf. *dalliana* (Frierson, 1913)	Myanmar: Ayeyarwady River basin, Mali Hka River basin, Nam Balak River	RMBH biv0102_7	KX865899	KX865660	KX865768
Yaukthwa zayleymanensis (Preston, 1912)	Myanmar: Ayeyarwady River basin, Bani river	RMBH biv0665_1	MN275073	MN307260	MN307202
Yaukthwa zayleymanensis (Preston, 1912)	Myanmar: Ayeyarwady River basin, Tarkat Stream	RMBH biv0679_1	MN275074	MN307261	MN307203
Yaukthwa zayleymanensis (Preston, 1912)	Myanmar: Ayeyarwady River basin, Tarkat Stream	RMBH biv0679_2	MN275075	MN307262	MN307204
Balwantia elongatula (Bolotov et al., 2019) **comb. nov.**	Myanmar: Ayeyarwady River basin, Chindwin River	RMBH biv0346_2	MK372407	MK372455	MK372485
Balwantia elongatula (Bolotov et al., 2019) **comb. nov.**	Myanmar: Ayeyarwady River basin, Chindwin River	RMBH biv0346_3	MK372408	MK372456	MK372486
Balwantia elongatula (Bolotov et al., 2019) **comb. nov.**	Myanmar: Ayeyarwady River basin, Myit Tha (Manipur) River	RMBH biv0341_2	MK372400	MK372451	MK372480
Balwantia baniensis **sp. nov.**	Myanmar: Ayeyarwady River basin, Bani River	RMBH biv0666_1	MN275076	MN307263	MN307205
Balwantia baniensis **sp. nov.**	Myanmar: Ayeyarwady River basin, Bani River	RMBH biv0666_2	MN275077	MN307264	MN307206
Balwantia baniensis **sp. nov.**	Myanmar: Ayeyarwady River basin, Bani River	RMBH biv0666_3	MN275078	MN307265	MN307207
**RECTIDENTINI Modell, 1942**					
Ensidens pazii (Lea, 1862)	Laos: Mekong River basin	NCSM 84889	KX822641	n/a	KX822598
Ensidens pazii (Lea, 1862)	Thailand: Chao Phraya River basin	E27A	MG025627	n/a	n/a
Ensidens pazii (Lea, 1862)	Thailand: Chao Phraya River basin	E36	MG025639	n/a	n/a
Ensidens sp.1	Thailand: Mekong River basin	E23A	MG025621	n/a	n/a
Ensidens sp.1	Thailand: Mekong River basin	E50	MG025657	n/a	n/a
Ensidens sp.1	Thailand: Mekong River basin	E64A	MG025684	n/a	n/a
Ensidens sp.2	Laos: Mekong River basin	NCSM 84902	KX822642	n/a	KX822599

Species	Locality	Specimen Voucher	NCBI's GenBank acc. nos.		
			COI	*16S rRNA*	*28S rRNA*
Ensidens sp.2	Thailand: Mekong River basin	E64	MG025677	n/a	n/a
Ensidens sp.2	Thailand: Mekong River basin	E64B	MG025685	n/a	n/a
Ensidens ingallsianus (Lea, 1852)	Cambodia: Mekong River basin	UMMZ 304651	KP795033	KP795053	KP795015
Ensidens ingallsianus (Lea, 1852)	Thailand: Mekong River basin, Tonle Sap	E54	MG025661	n/a	n/a
Ensidens ingallsianus (Lea, 1852)	Thailand: Mekong River basin, Bang Prakong River	E07	MG025674	n/a	n/a
Ensidens sagittarius (Lea, 1856)	Thailand: Mekong River basin	E63	MG025673	n/a	n/a
Ensidens sagittarius (Lea, 1856)	Thailand: Mekong River basin	E59	MG025668	n/a	n/a
Ensidens sagittarius (Lea, 1856)	Thailand: Mekong River basin	E57	MG025664	n/a	n/a
Ensidens sp. 'Thai'	Thailand: Mekong River basin, artificial pond near the Ban Nong-Bua village	RMBH biv0117_1	KX865942	KX865690	KX865813
Ensidens sp. 'Thai'	Thailand: Mekong River basin, artificial pond near the Ban Nong-Bua village	RMBH biv0117_2	KX865943	KX865691	KX865814
Ensidens sp. 'Thai'	Thailand: Mekong River basin, artificial pond near the Ban Nong-Bua village	RMBH biv0117_3	KX865944	KX865692	KX865815
Ensidens sp. 'Mun'	Thailand: Mekong River basin, Chi River	RMBH biv0128_1	KX865945	KX865693	KX865816
Ensidens sp. 'Mun'	Thailand: Mekong River basin, Chi River	RMBH biv0128_2	KX865946	KX865694	KX865817
Ensidens sp. 'Mun'	Thailand: Mekong River basin, Chi River	RMBH biv0123_2	KX865949	KX865695	KX865820
Hyriopsis gracilis Haas, 1910	Thailand: Mekong River basin, Chi River	RMBH biv0130_1	KX865951	KX865697	KX865822
Hyriopsis gracilis Haas, 1910	Thailand: Mekong River basin, Chi River	RMBH biv0130_2	KX865952	KX865698	KX865823
Hyriopsis gracilis Haas, 1910	Thailand: Mekong River basin, Chi River	RMBH biv0130_3	KX865953	n/a	KX865824
Hyriopsis sp.1	Thailand: Chao Phraya River basin	839512HbB	KX383948	n/a	n/a
Hyriopsis bialatus Simpson, 1900	Malaysia	BIV1775	KX051273	n/a	n/a
Hyriopsis myersiana (Lea, 1856)	Thailand	n/a	KX822645	n/a	KX822602
Hyriopsis desowitzi Brandt, 1974	Thailand	n/a	KX822644	n/a	KX822601
Rectidens sumatrensis (Dunker, 1852)	Malaysia	n/a	KX822664	n/a	KX822620
Rectidens sumatrensis (Dunker, 1852)	Malaysia	RMBH biv0211_1	MF352288	n/a	MF352405
**GONIDEINI s. str.**					

Species	Locality	Specimen Voucher	NCBI's GenBank acc. nos.		
			COI	*16S rRNA*	*28S rRNA*
Solenaia oleivora (Heude, 1877) sp.1	China	16_NCU_XPWU_SU181	MG463091	n/a	MG595618
Solenaia oleivora (Heude, 1877) sp.2	China	16_NCU_XPWU_SU182	MG463092	n/a	MG595619
Solenaia oleivora (Heude, 1877) sp.3	China	16_NCU_XPWU_SU183	MG463093	n/a	MG595620
Solenaia sp.	Vietnam	RMBH biv0089_1	KY561639	KY561653	KY561670
Solenaia carinata (Heude, 1877)	China	n/a	KX822669	NC_023250	KX822626
Leguminaia wheatleyi (Lea, 1862)	Turkey: Karasu River	RMBH biv0177_7	MN402614	MN396725	MN396721
Microcondylaea bonellii (Férussac, 1827)	Italy	n/a	KX822652	KP218021	KX822609
Gonidea angulata (Lea, 1838)	USA: Okanagan Lake	RMBH biv0294_1	MN402615	MN396726	MN396722
Ptychorhynchus pfisteri (Heude, 1874)	China	16_NCU_XPWU_SU127	MG463036	n/a	MG595564
Parvasolenaia rivularis (Heude, 1877)	China	16_NCU_XPWU_SU190	MG463101	n/a	MG595627
**LAMPROTULINI Modell, 1942**					
Lamprotula leaii (Griffith & Pidgeon, 1833)	Vietnam	RMBH biv0200_1	MN402616	MN396727	MN396723
Lamprotula sp.	China	16_NCU_XPWU_SU092	MG463001	n/a	MG595529
Lamprotula caveata (Heude, 1877)	China	16_NCU_XPWU_SU081	MG462991	n/a	MG595518
Potomida littoralis (Cuvier, 1798)	Turkey: Karasu River	RMBH biv0177_10	MN402617	MN396728	MN396724
Pronodularia japanensis (Lea, 1859)	Japan	NCSM 27183	KX822659	AB055625	KX822615
**CHAMBERLAININI Bogan, Froufe & Lopes-Lima, 2017**					
Sinochyriopsis cumingii (Lea, 1852)	China	16_NCU_XPWU_SU177	NC_011763	NC_011763	MG595614
Chamberlainia hainesiana (Lea, 1856)	Thailand	BIV46	KX822635	n/a	KX822592
**PARREYSIINAE Henderson 1935**					
**INDOCHINELLINI Bolotov et al., 2018**					
Indochinella pugio pugio (Benson, 1862)	Myanmar: Ayeyarwady River basin, Lake Nant Phar	RMBH biv0258_1	MF352261	MF352325	MF352386
Indochinella pugio pugio (Benson, 1862)	Myanmar: Ayeyarwady River basin, Lake Nant Phar	RMBH biv0258_2	MF352262	MF352326	MF352389
Indochinella pugio pugio (Benson, 1862)	Myanmar: Ayeyarwady River basin, Chindwin River, Pauk In Lake	UA 20739	JN243899	KP795046	JN243879
Indochinella pugio viridissima Bolotov et al.,	Myanmar: Sittaung River basin, Myit Kyi Pauk	RMBH biv0251/1	MF352242	MF352312	MF352370

Species	Locality	Specimen Voucher	NCBI's GenBank acc. nos.		
			COI	*16S rRNA*	*28S rRNA*
2019	Stream				
Indochinella pugio viridissima Bolotov et al., 2019	Myanmar: Bilin River	RMBH biv0371_3	MK372428	n/a	n/a
Indochinella pugio viridissima Bolotov et al., 2019	Myanmar: Bago River	RMBH biv0381_3	MK372434	n/a	n/a
Indochinella pugio daweiensis Bolotov et al., 2019	Myanmar: Dawei River	RMBH biv0147_3	KX865852	KX865623	KX865724
Indochinella pugio daweiensis Bolotov et al., 2019	Myanmar: Dawei River	RMBH biv0148_7	KX865856	KX865627	KX865728
Indochinella pugio daweiensis Bolotov et al., 2019	Myanmar: Dawei River	RMBH biv0148_15	KX865857	KX865628	KX865729
Indonaia caerulea (Lea, 1831)	India: Krishna River basin, fish pond	RRc1	KT869141	n/a	n/a
Indonaia andersoniana (Nevill, 1877)	Myanmar: Ayeyarwady River basin, Lake Indawgyi	RMBH biv0108/1	KX865835	KX865606	KX865709
Indonaia andersoniana (Nevill, 1877)	Myanmar: Ayeyarwady River basin, Lake Nant Phar	RMBH biv0259_1	MF352263	MF352327	MF352387
Indonaia andersoniana (Nevill, 1877)	Myanmar: Ayeyarwady River basin, Nant Sa Yi River	RMBH biv0253_1	MF352275	MF352337	MF352397
Indonaia aff. *khadakvaslaensis* (Ray, 1966)	India	SBM7	KF690124	n/a	n/a
Indonaia aff. *khadakvaslaensis* (Ray, 1966)	India	SBM19	KF690122	n/a	n/a
Indonaia aff. *khadakvaslaensis* (Ray, 1966)	India	SBM23	KF690123	n/a	n/a
Indonaia lima (Simpson, 1900)	India: Krishna River, Nagarjuna Sagar	RRl1	KP268827	n/a	n/a
Indonaia subclathrata (Martens, 1899)	Myanmar: Ayeyarwady River basin, Chindwin River	RMBH biv0347/1	MH700480	MN307266	MH697870
Indonaia subclathrata (Martens, 1899)	Myanmar: Ayeyarwady River basin, Chindwin River	RMBH biv0347/2	MN275079	n/a	MN307208
Indonaia subclathrata (Martens, 1899)	Myanmar: Bani River	biv667_1	MN275080	n/a	n/a
Radiatula mouhoti Vikhrev, Bolotov & Konopleva, 2017	Myanmar: Sittaung River near Taungoo	RMBH biv0248_1	MF352234	MF352305	MF352363
Radiatula mouhoti Vikhrev, Bolotov & Konopleva, 2017	Myanmar: Sittaung River near Taungoo	RMBH biv0248_4	MF352236	MF352306	MF352364
Radiatula mouhoti Vikhrev, Bolotov & Konopleva, 2017	Myanmar: Sittaung River near Taungoo	RMBH biv0253_1	MF352248	MF352317	MF352375
Radiatula chaudhurii	Myanmar: Ayeyarwady	RMBH biv0260_5	MF352266	MF352330	MF352390

Species	Locality	Specimen Voucher	NCBI's GenBank acc. nos.		
			COI	*16S rRNA*	*28S rRNA*
(Preston, 1912)	River basin, Tar Pein River				
Radiatula chaudhurii (Preston, 1912)	Myanmar: Ayeyarwady River basin, Tar Pein River	RMBH biv0260_10	MF352268	MF352332	MF352392
Radiatula chaudhurii (Preston, 1912)	Myanmar: Ayeyarwady River basin, Tar Pein River	RMBH biv0260_9	MF352267	MF352331	MF352391
Radiatula myitkyinae (Prashad, 1930)	Myanmar: Ayeyarwady River basin, Lake Indawgyi	RMBH biv0107_1	KX865838	KX865609	KX865710
Radiatula myitkyinae (Prashad, 1930)	Myanmar: Ayeyarwady River basin, Lake Indawgyi	RMBH biv0106_3	KX865842	KX865614	KX865714
Radiatula myitkyinae (Prashad, 1930)	Myanmar: Ayeyarwady River basin, Lake Indawgyi	RMBH biv0106_2	KX865843	KX865613	KX865715
Radiatula crispisulcata (Benson, 1862)	Myanmar: Ayeyarwady River basin, Nga Wun River	RMBH biv0671_1	MN275081	MN307267	MN307209
Radiatula crispisulcata (Benson, 1862)	Myanmar: Ayeyarwady River basin, Nga Wun River	RMBH biv0671_2	MN275082	MN307268	MN307210
Radiatula crispisulcata (Benson, 1862)	Myanmar: Ayeyarwady River basin, Nga Wun River	RMBH biv0671_3	MN275083	MN307269	MN307211
Scabies humilis (Lea, 1856)	Thailand: Mekong River basin, Chi River	RMBH biv0126/1	KX865844	KX865615	KX865716
Scabies humilis (Lea, 1856)	Thailand: Khorat Plateau	UF 507600 (2014-0570)	MH350923	n/a	MH350923
Scabies humilis (Lea, 1856)	Thailand: Khorat Plateau	UF 507616 (2014-0604)	MH350925	n/a	MH350974
Scabies phaselus (Lea, 1856)	Thailand: Mekong River basin, Chi River	RMBH biv0124/1	KX865850	KX865621	KX865722
Scabies phaselus (Lea, 1856)	Thailand: Khorat Plateau	UF 507608 (2014-0582)	MH350924	n/a	MH350973
Scabies phaselus (Lea, 1856)	Thailand: Khorat Plateau	UF 507651 (2014-0683)	MH350927	n/a	MH350976
Scabies crispata (Gould, 1843)	Thailand: Chao Phraya River basin	UF 507682 (2012-0409)	MH350920	n/a	MH350969
Scabies crispata (Gould, 1843)	Thailand: Mae Klong River	UF 507693 (2012-0428)	MH350921	n/a	MH350970
Scabies crispata (Gould, 1843)	Thailand: Mae Klong River	UF 507590 (2014-0030)	MH350922	n/a	MH350971
Scabies nucleus (Lea, 1856)	Thailand: Khorat Plateau	UF 507738 (ICH-00706)	MH350943	n/a	MH350992
Scabies nucleus (Lea, 1856)	Thailand: Khorat Plateau	UF 507738 (ICH-00707)	MH350944	n/a	MH350993
Scabies pilata (Lea, 1866)	Thailand: Khorat Plateau	UF 507760 (ICH-00740)	MH350945	n/a	MH350994
Scabies pilata (Lea, 1866)	Thailand: Khorat Plateau	UF 507752 (ICH-00743)	MH350946	n/a	MH350995
Scabies pilata (Lea, 1866)	Thailand: Khorat Plateau	UF 507761 (ICH-00754)	MH350947	n/a	MH350996

Species	Locality	Specimen Voucher	NCBI's GenBank acc. nos.		
			COI	*16S rRNA*	*28S rRNA*
Scabies anceps (Deshayes in Deshayes & Jullien, 1874)	Cambodia: Kratie – Stung Treng	UF 507394 (ICH-00456)	MH350929	n/a	MH350978
Scabies anceps (Deshayes in Deshayes & Jullien, 1874)	Mekong Delta	UF 507431 (ICH-00583)	MH350936	n/a	MH350985
Scabies anceps (Deshayes in Deshayes & Jullien, 1874)	Kratie – Stung Treng	UF 507385 (ICH-00428)	MH350928	n/a	MH350928
Scabies mandarinus (Morelet, 1863)	Mekong Delta	UF 507450 (ICH-00630)	MH350939	n/a	MH350988
Scabies mandarinus (Morelet, 1863)	Mekong Delta	UF 507434 (ICH-00593)	MH350937	n/a	MH350986
Scabies mandarinus (Morelet, 1863)	Cambodia: Mekong River basin	UMMZ:304646	KP795023	KP795048	KP795006
Scabies scobinatus (Lea, 1856)	Thailand: Khorat Plateau	UF 507782 (ICH-00796)	MH350951	n/a	MH351000
Scabies scobinatus (Lea, 1856)	Thailand: Khorat Plateau	UF 507825 (ICH-00879)	MH350958	n/a	MH351007
Scabellus songkramensis (Kongim & Panha, 2015) **gen. & comb. nov.**	Thailand: Khorat Plateau	UF 507634 (2014-0658)	MH350926	n/a	MH350975
Scabellus songkramensis (Kongim & Panha, 2015) **gen. & comb. nov.**	Thailand: Khorat Plateau	UF 507775 (ICH-00768)	MH350948	n/a	MH350997
Scabellus songkramensis (Kongim & Panha, 2015) **gen. & comb. nov.**	Thailand: Khorat Plateau	UF 507766 (ICH-00779)	MH350949	n/a	MH350998
Unionetta fabagina (Deshayes in Deshayes & Jullien, 1874)	Cambodia: Kratie – Stung Treng	UF 507399 (ICH-00489)	MH350930	n/a	MH350979
Unionetta fabagina (Deshayes in Deshayes & Jullien, 1874)	Cambodia: Kratie – Stung Treng	UF 507399 (ICH-00490)	MH350931	n/a	MH350980
Unionetta fabagina (Deshayes in Deshayes & Jullien, 1874)	Cambodia: Kratie – Stung Treng	UF 507399 (ICH-00491)	MH350932	n/a	MH350981
Harmandia somboriensis Rochebrune, 1881	Thailand: Khorat Plateau	UF 507791 (ICH-00810)	MH350952	n/a	MH351001
Harmandia somboriensis Rochebrune, 1881	Thailand: Khorat Plateau	UF 507791 (ICH-00811)	MH350953	n/a	MH351002
Harmandia somboriensis Rochebrune, 1881	Thailand: Khorat Plateau	UF 507831 (ICH-00889)	MH350959	n/a	MH351008
**LAMELLIDENTINI Modell, 1942**					
Trapezidens exolescens (Gould, 1843)	Myanmar: Dawei River	RMBH biv0145_3	KX230532	KX230548	KX230559
Trapezidens exolescens (Gould, 1843)	Myanmar: Dawei River	RMBH biv0145_12	KX230535	KX230549	KX230560
Trapezidens exolescens (Gould, 1843)	Myanmar: Dawei River	RMBH biv0145_23	KX230542	KX230542	KX230564
Trapezidens scutum (Sowerby, 1868)	Myanmar: Tanintharyi River basin, Nga Wun River	RMBH biv0630_1	MN275084	MN307270	MN307212
Trapezidens scutum	Myanmar: Tanintharyi	RMBH biv0630_2	MN275085	MN307271	MN307213

Species	Locality	Specimen Voucher	NCBI's GenBank acc. nos.		
			COI	***16S rRNA***	***28S rRNA***
(Sowerby, 1868)	River basin, Nga Wun River				
Trapezidens scutum (Sowerby, 1868)	Myanmar: Tanintharyi River basin, Nga Wun River	RMBH biv0632_1	MN275086	MN307272	MN307214
Trapezidens angustior (Hanley & Theobald, 1876)	Myanmar: Sittaung River basin, Myit Kyi Pauk Stream	RMBH biv0250_8	MF352240	MF352310	MF352310
Trapezidens angustior (Hanley & Theobald, 1876)	Myanmar: Bilin River	RMBH biv0370_1	MN275087	MN307273	MN307215
Trapezidens angustior (Hanley & Theobald, 1876)	Myanmar: Chain Stream	RMBH biv0394_1	MN275088	n/a	n/a
Trapezidens dolichorhynchus (Tapparone Canefri, 1889)	Myanmar: Ayeyarwady River	UA 20729.1	JN243903	KP795042	JN243881
Trapezidens dolichorhynchus (Tapparone Canefri, 1889)	Myanmar: Ayeyarwady River	RMBH biv0417_1	MN275089	MN307274	MN307216
Trapezidens dolichorhynchus (Tapparone Canefri, 1889)	Myanmar: Ayeyarwady River basin, Myit Tha (Manipur) River	RMBH biv0336_3	MN275090	n/a	MN307217
Lamellidens savadiensis (Nevill, 1877)	Myanmar: Ayeyarwady River basin, Lake Indawgyi	RMBH biv0109_1	KX230544	KX230555	KX230566
Lamellidens savadiensis (Nevill, 1877)	Myanmar: Ayeyarwady River basin, Lake Indawgyi	RMBH biv0109_2	KX230545	KX230556	KX230567
Lamellidens savadiensis (Nevill, 1877)	Myanmar: Ayeyarwady River basin, Lake Indawgyi	RMBH biv0109_3	KX230546	KX230557	KX230568
Lamellidens generosus (Gould, 1847)	Myanmar: Sittaung River basin, Pathi river	RMBH biv0242_3	MF352219	MF352293	MF352351
Lamellidens generosus (Gould, 1847)	Myanmar: Sittaung River basin, Pathi river	RMBH biv0243_10	MF352222	MF352296	MF352354
Lamellidens generosus (Gould, 1847)	Myanmar: Sittaung River basin, reservoir of Yetho River	RMBH biv0244_3	MF352226	MF352298	MF352356
Lamellidens ferrugineus (Annandale, 1918)	Myanmar: Salween River basin, lower channel of Lake Inle	RMBH biv0113_8	KX865825	KX865596	KX865699
Lamellidens ferrugineus (Annandale, 1918)	Myanmar: Salween River basin, lower channel of Lake Inle	RMBH biv0113_12	KX865826	KX865597	KX865700
Lamellidens ferrugineus (Annandale, 1918)	Myanmar: Salween River basin, lower channel of Lake Inle	RMBH biv0113_14	KX865827	KX865598	KX865701
Lamellidens marginalis (Lamarck, 1819)	Myanmar: Kaladan River basin, unnamed puddle	RMBH biv0153	KX230547	KX230558	KX230569
**PARREYSIINI Henderson, 1935**					
Parreysia cf. *corrugata*	India	RNB1	KJ872809	n/a	n/a

Species	Locality	Specimen Voucher	NCBI's GenBank acc. nos.		
			COI	*16S rRNA*	*28S rRNA*
(Müller, 1774) sp.1					
Parreysia cf. *corrugata* (Müller, 1774) sp.1	India	RNB2	KJ872810	n/a	n/a
Parreysia cf. *corrugata* (Müller, 1774) sp.1	India	RPc10	KT869140	n/a	n/a
Parreysia cf. *corrugata* (Müller, 1774) sp.2	India	RNBI	JQ861229	n/a	n/a
Parreysia cf. *corrugata* (Müller, 1774) sp.2	India	RNBI5	JQ861234	n/a	n/a
Parreysia cf. *corrugata* (Müller, 1774) sp.3	India	RNBI11	JQ861232	n/a	n/a
Parreysia cf. *corrugata* (Müller, 1774) sp.3	India	RNBI13	JQ861231	n/a	n/a
Parreysia cf. *corrugata* (Müller, 1774) sp.3	India	RNBI14	JQ861230	n/a	n/a
Parreysia cf. *favidens* (Müller, 1774) sp.1	India	RNBI16	JQ861241	n/a	n/a
Parreysia cf. *favidens* (Müller, 1774) sp.1	India	RNBI17	JQ861240	n/a	n/a
Parreysia cf. *favidens* (Müller, 1774) sp.1	India	RNBI18	JQ861239	n/a	n/a
Parreysia cf. *favidens* (Müller, 1774) sp.2	India	SBM3	KF690110	n/a	n/a
Parreysia rakhinensis **sp. nov.**	Myanmar: Kyeintali Stream, upstream of Ohtein village	RMBH biv0652_1	MN275091	MN307275	MN307218
Parreysia rakhinensis **sp. nov.**	Myanmar: Kyeintali Stream, upstream of Ohtein village	RMBH biv0652_2	MN275092	n/a	n/a
Parreysia rakhinensis **sp. nov.**	Myanmar: Kyeintali Stream, upstream of Ohtein village	RMBH biv0652_3	MN275093	n/a	n/a
Parreysia rakhinensis **sp. nov.**	Myanmar: Kyeintali Stream, upstream of Ohtein village	RMBH biv0652_4	MN275094	n/a	n/a
Parreysia rakhinensis **sp. nov.**	Myanmar: Kyeintali Stream, upstream of Ohtein village	RMBH biv0652_6	MN275095	n/a	n/a
Parreysia rakhinensis **sp. nov.**	Myanmar: Sa Lu Stream	RMBH biv0653_1	MN275096	n/a	n/a
Parreysia rakhinensis **sp. nov.**	Myanmar: Sa Lu Stream	RMBH biv0653_2	MN275097	n/a	n/a
Parreysia rakhinensis **sp. nov.**	Myanmar: Sa Lu Stream	RMBH biv0653_3	MN275098	n/a	n/a
Parreysia rakhinensis **sp. nov.**	Myanmar: Sa Lu Stream	RMBH biv0654_1	MN275099	n/a	n/a
Parreysia rakhinensis **sp. nov.**	Myanmar: Sa Lu Stream	RMBH biv0654_2	MN275100	n/a	n/a
Parreysia rakhinensis **sp. nov.**	Myanmar: Thandwe River, Ywar Shai village	RMBH biv0655_1	MN275101	n/a	n/a
Parreysia rakhinensis **sp. nov.**	Myanmar: Thandwe River, Ywar Shai village	RMBH biv0655_2	MN275102	n/a	n/a
Parreysia rakhinensis **sp. nov.**	Myanmar: Thandwe River, Ywar Shai village	RMBH biv0655_3	MN275103	n/a	n/a

Species	Locality	Specimen Voucher	NCBI's GenBank acc. nos.		
			COI	*16S rRNA*	*28S rRNA*
Parreysia rakhinensis **sp. nov.**	Myanmar: Thandwe River, Ywar Shai village	RMBH biv0656	MN275104	MN307276	MN307219
Parreysia rakhinensis **sp. nov.**	Myanmar	RMBH biv0657_1	MN275105	n/a	n/a
Parreysia rakhinensis **sp. nov.**	Myanmar	RMBH biv0657_2	MN275106	n/a	n/a
Parreysia rakhinensis **sp. nov.**	Myanmar	RMBH biv0657_3	MN275107	n/a	n/a
Parreysia rakhinensis **sp. nov.**	Myanmar: Toungup River	RMBH biv0658_1	MN275108	n/a	n/a
Parreysia rakhinensis **sp. nov.**	Myanmar: Toungup River	RMBH biv0658_2	MN275109	n/a	n/a
Parreysia rakhinensis **sp. nov.**	Myanmar: Toungup River	RMBH biv0658_3	MN275110	n/a	n/a
Parreysia rakhinensis **sp. nov.**	Myanmar: Ann River	RMBH biv0659_1	MN275111	n/a	n/a
Parreysia rakhinensis **sp. nov.**	Myanmar: Ann River	RMBH biv0659_2	MN275112	n/a	n/a
Parreysia rakhinensis **sp. nov.**	Myanmar: Ann River	RMBH biv0659_3	MN275113	n/a	n/a
Parreysia rakhinensis **sp. nov.**	Myanmar: tributary of the Ann River	RMBH biv0660_1	MN275114	n/a	n/a
Parreysia rakhinensis **sp. nov.**	Myanmar: tributary of the Ann River	RMBH biv0660_2	MN275115	n/a	n/a
Parreysia rakhinensis **sp. nov.**	Myanmar: tributary of the Ann River	RMBH biv0660_3	MN275116	MN307277	MN307220
**LEOPARREYSIINI Vikhrev, Bolotov & Kondakov, 2017**					
Leoparreysia olivacea (Prashad, 1930)	Myanmar: Ayeyarwady River, 3 mi SW Pakkoku, Magway Division	UMMZ 304641	KP795022	KP795044	KP795005
Leoparreysia canefrii Vikhrev, Bolotov & Kondakov, 2017	Myanmar: Sittaung River near Taungoo	RMBH biv0249	MF352237	MF352307	MF352365
Leoparreysia canefrii Vikhrev, Bolotov & Kondakov, 2017	Myanmar: Sittaung River near Taungoo	RMBH biv0252_1	MF352245	MF352315	MF352373
Leoparreysia canefrii Vikhrev, Bolotov & Kondakov, 2017	Myanmar: Sittaung River near Taungoo	RMBH biv0252_2	MF352246	MF352316	MF352374
Leoparreysia burmana (Blanford, 1869)	Myanmar: Ayeyarwady River	RMBH biv0424_3	MK372443	MK372474	MK372506
Leoparreysia burmana (Blanford, 1869)	Myanmar: Ayeyarwady River	RMBH biv0419_3	MK372438	MK372470	MK372502
Leoparreysia burmana (Blanford, 1869)	Myanmar: Ayeyarwady River basin, Chindwin River	RMBH biv0350_1	MK372412	MK372460	MK372490
Leoparreysia tavoyensis (Gould, 1843)	Myanmar: Dawei River	RMBH biv0149	KX230543	KX230554	KX230565
Leoparreysia tavoyensis (Gould, 1843)	Myanmar: Haungthayaw River	RMBH biv0362_2	MK372424	n/a	n/a
Leoparreysia tavoyensis (Gould, 1843)	Myanmar: Haungthayaw River	RMBH biv0362_3	MK372425	n/a	n/a

Species	Locality	Specimen Voucher	NCBI's GenBank acc. nos.		
			COI	*16S rRNA*	*28S rRNA*
Leoparreysia whitteni Bolotov et al., 2019	Myanmar: Ayeyarwady River basin, Chindwin River	RMBH biv0349	MK372411	MK372459	MK372489
Leoparreysia whitteni Bolotov et al., 2019	Myanmar: Ayeyarwady River	RMBH biv0435	MK372446	MK372475	MK372507
Leoparreysia whitteni Bolotov et al., 2019	Myanmar: Ayeyarwady River	RMBH biv0449	MK372449	MK372476	MK372508
**COELATURINI Modell, 1942**					
Nitia teretiuscula (Philippi, 1847)	Egypt: Nile River	n/a	JN243897	n/a	JN243875
Coelatura aff. *aegyptiaca* (Cailliaud, 1827)	Egypt: Nile River	n/a	JN243894	KP795045	JN243872
**Outgroup Taxa**					
**MARGARITIFERIDAE Henderson, 1929**					
Gibbosula laosensis (Lea, 1863)	Laos: Mekong River basin, Nam Long River	RMBH biv0186_1	JX497731	KC845943	KT343741
Margaritifera dahurica (Middendorff,1850)	Far East of Russia: Amur River basin, Ilistaya River	RMBH biv0092_6	KJ161516	KJ943526	KT343747
Margaritifera margaritifera (Linnaeus, 1758)	Northwestern Russia: Onega River basin, Somba River	RMBH biv0618	KX550089	KX550091	KX550093
Margaritifera laevis (Haas, 1910)	Far East of Russia: Kurile Archipelago, Kunashir Island, Sennaya River	RMBH biv0036_22	KJ161500	KJ943523	KT343742
Margaritifera middendorffi (Rosén, 1926)	Far East of Russia: Kamchatka, Bolshaya River basin, Nachilova River	RMBH biv0099_6	KJ161547	KJ943528	KT343745
**IRIDINIDAE Swainson, 1840**					
Aspatharia pfeifferiana (Bernardi, 1860)	Zambia: Chambeshi River	BIVAToL-330	KC429107	KC429264	n/a
Chambardia wahlbergi (Krauss, 1848)	Zambia: Zambezi River	ANSP 419403	JN243886	KP184845	JN243864
**ETHERIIDAE Deshayes, 1832**					
Etheria elliptica Lamarck, 1807	Zambia: Chambeshi River	FMNH 343390	KP184897	KP184847	KP184873
**MYCETOPODIDAE Gray, 1840**					
Anodontites elongata (Swainson, 1823)	Peru	FMNH 343931	KP184896	KP184846	KP184872
**HYRIIDAE Swainson, 1840**					
Triplodon corrugatus (Lamarck, 1819)	Peru	ANSP 416338	JN243890	KP184851	JN243868
Castalia ambigua Lamarck, 1819	Peru	ANSP 416341	JN243889	KP184848	JN243867
Microdontia anodontaeformis (Tapparone Canefri, 1883)	Guyana	UMMZ 304509	KP184909	KP184861	KP184885

Species	Locality	Specimen Voucher	NCBI's GenBank acc. nos.		
			COI	***16S rRNA***	***28S rRNA***
Alathyria jacksoni Iredale, 1934	New Guinea	UMMZ 304512	KP184912	KP184864	KP184888
Alathyria pertexta Iredale, 1934	Australia: New South Wales	UMMZ 304510	KP184910	KP184862	KP184886
Alathyria profuga (Gould, 1850)	Australia: Queensland	UMMZ 304513	KP184913	KP184865	KP184889
Lortiella froggatti Iredale, 1934*	Australia: New South Wales	n/a	AF231746	KP184867	KP184891
Velesunio ambiguus (Philippi, 1847)	Western Australia	FMNH 337195	KP184915	KP184868	KP184892
**TRIGONIIDAE Lamarck, 1819**					
Neotrigonia margaritacea (Lamarck, 1804)*	Tasmania and Australia	n/a	U56850	DQ280034	DQ279963
Neotrigonia lamarckii (Gray, 1838)	Australia: Coral Sea, North Stradbroke Island, Queensland	BIVAToL-97	KC429105	KC429262	KC429443

*Chimeric sequences. n/a – not available.

Supplementary Table 2. Shell measurements and reference DNA sequences for the type series of new freshwater mussel species (Unionidae) from Southeast Asia

Taxon	Status of Specimen	Specimen Voucher*	Shell measurements (mm)			NCBI's GenBank acc. nos.		
			SL	SH	SW	*COI*	*16S rRNA*	*28S rRNA*
Parreysia rakhinensis **sp. nov.**	Holotype	RMBH biv0652_1	58.7	33.7	19.6	MN275091	MN307275	MN307218
P. rakhinensis **sp. nov.**	Paratype	RMBH biv0652_2	54.9	29.0	17.9	MN275092	n/a	n/a
P. rakhinensis **sp. nov.**	Paratype	RMBH biv0652_3	44.0	25.0	16.3	MN275093	n/a	n/a
P. rakhinensis **sp. nov.**	Paratype	RMBH biv0652_4	46.3	25.8	14.7	MN275094	n/a	n/a
P. rakhinensis **sp. nov.**	Paratype	RMBH biv0652_6	52.2	31.0	17.3	MN275095	n/a	n/a
P. rakhinensis **sp. nov.**	Paratype	RMBH biv0653_1	49.2	31.1	20.2	MN275096	n/a	n/a
P. rakhinensis **sp. nov.**	Paratype	RMBH biv0653_2	52.1	34.6	23.3	MN275097	n/a	n/a
P. rakhinensis **sp. nov.**	Paratype	RMBH biv0653_3	54.6	36.8	22.9	MN275098	n/a	n/a
P. rakhinensis **sp. nov.**	Paratype	RMBH biv0654_1	44.8	30.2	20.8	MN275099	n/a	n/a
P. rakhinensis **sp. nov.**	Paratype	RMBH biv0654_2	31.7	20.8	12.4	MN275100	n/a	n/a
P. rakhinensis **sp. nov.**	Paratype	RMBH biv0655_1	45.2	28.2	16.6	MN275101	n/a	n/a
P. rakhinensis **sp. nov.**	Paratype	RMBH biv0655_2	43.9	29.1	15.9	MN275102	n/a	n/a
P. rakhinensis **sp. nov.**	Paratype	RMBH biv0655_3	48.5	30.1	17.0	MN275103	n/a	n/a
P. rakhinensis **sp. nov.**	Paratype	RMBH biv0656	41.2	23.2	14.0	MN275104	MN307276	MN307219
P. rakhinensis **sp. nov.**	Paratype	RMBH biv0657_1	29.3	18.5	10.3	MN275105	n/a	n/a
P. rakhinensis **sp. nov.**	Paratype	RMBH biv0657_2	37.3	22.2	13.8	MN275106	n/a	n/a
P. rakhinensis **sp. nov.**	Paratype	RMBH biv0657_3	37.6	23.7	13.0	MN275107	n/a	n/a
P. rakhinensis **sp. nov.**	Paratype	RMBH biv0658_1	53.0	34.3	19.5	MN275108	n/a	n/a
P. rakhinensis **sp. nov.**	Paratype	RMBH biv0658_2	55.8	35.2	21.5	MN275109	n/a	n/a
P. rakhinensis **sp. nov.**	Paratype	RMBH biv0658_3	51.1	33.3	20.6	MN275110	n/a	n/a
P. rakhinensis **sp. nov.**	Paratype	RMBH biv0659_1	46.5	31.3	18.9	MN275111	n/a	n/a
P. rakhinensis **sp. nov.**	Paratype	RMBH biv0659_2	30.9	20.5	12.6	MN275112	n/a	n/a
P. rakhinensis **sp. nov.**	Paratype	RMBH biv0659_3	62.3	41.7	24.2	MN275113	n/a	n/a
P. rakhinensis **sp. nov.**	Paratype	RMBH biv0660_1	41.5	27.4	15.8	MN275114	n/a	n/a
P. rakhinensis **sp. nov.**	Paratype	RMBH biv0660_2	39.2	25.6	15.9	MN275115	n/a	n/a
P. rakhinensis **sp. nov.**	Paratype	RMBH biv0660_3	40.6	25.9	15.5	MN275116	MN307277	MN307220
Balwantia baniensis **sp. nov.**	Holotype	RMBH biv0666_2	57.0	23.2	15.3	MN275077	MN307264	MN307206
B. baniensis **sp. nov.**	Paratype	RMBH biv0666_1	46.1	19.5	12.7	MN275076	MN307263	MN307205
B. baniensis **sp. nov.**	Paratype	RMBH biv0666_3	50.8	21.7	12.7	MN275078	MN307265	MN307207
B. baniensis **sp. nov.**	Paratype	RMBH biv0666_4	61.4	26.3	16.7	n/a	n/a	n/a
B. baniensis **sp. nov.**	Paratype	RMBH biv0666_5	55.1	22.8	13.8	n/a	n/a	n/a
Trapezoideus lenya **sp. nov.**	Holotype	RMBH biv0629_2	36.3	21.4	11.3	MN275068	MN307257	MN307198
T. lenya **sp. nov.**	Paratype	RMBH biv0629_1	33.1	19.1	10.2	MN275067	MN307256	MN307197
T. lenya **sp. nov.**	Paratype	RMBH biv0629_3	35.5	20.8	12.6	MN275069	MN307258	MN307199
T. lenya **sp. nov.**	Paratype	RMBH biv0629_5	35.7	21.1	10.9	n/a	n/a	n/a
Yaukthwa avaensis **sp. nov.**	Holotype	RMBH biv0680_3	38.3	20.8	14.1	MN275071	MN307259	MN307200
Y. avaensis **sp. nov.**	Paratype	RMBH biv0680_1	41.7	21.6	16.5	MN275070	n/a	n/a
Y. avaensis **sp. nov.**	Paratype	RMBH biv0680_5	37.0	21.0	14.7	MN275072	n/a	MN307201
Y. avaensis **sp. nov.**	Paratype	RMBH biv0680_2	46.6	25.1	17.0	n/a	n/a	n/a
Y. avaensis **sp. nov.**	Paratype	RMBH biv0680_4	38.5	22.7	14.7	n/a	n/a	n/a
Y. avaensis **sp. nov.**	Paratype	RMBH biv0680_6	39.8	22.2	17.0	n/a	n/a	n/a
Y. avaensis **sp. nov.**	Paratype	RMBH biv0680_7	38.8	21.6	12.6	n/a	n/a	n/a
Y. avaensis **sp. nov.**	Paratype	RMBH biv0680_8	35.1	20.0	14.0	n/a	n/a	n/a
Y. avaensis **sp. nov.**	Paratype	RMBH biv0680_9	37.0	21.0	14.6	n/a	n/a	n/a
Y. avaensis **sp. nov.**	Paratype	RMBH biv0680_10	24.7	14.1	8.7	n/a	n/a	n/a
Monodontina laosica **sp. nov.**	Holotype	UMMZ 304650	61.4	41.4	19.0	KP795029	KP795052	n/a
M. lenyanensis **sp. nov.**	Holotype	RMBH biv0628_2	63.4	40.1	23.7	MN275055	MN307246	MN307187
M. lenyanensis **sp. nov.**	Paratype	RMBH biv0628_1	52.4	32.1	17.2	MN275054	MN307245	MN307186
M. lenyanensis **sp. nov.**	Paratype	RMBH biv0628_3	59.1	38.0	19.8	MN275056	MN307247	MN307188
M. lenyanensis **sp. nov.**	Paratype	RMBH biv0628_4	60.7	37.6	21.7	n/a	n/a	n/a
M. lenyanensis **sp.**	Paratype	RMBH biv0628_5	57.3	36.5	18.7	n/a	n/a	n/a

Taxon	Status of Specimen	Specimen Voucher*	Shell measurements (mm)			NCBI's GenBank acc. nos.		
			SL	SH	SW	*COI*	*16S rRNA*	*28S rRNA*
**nov.**								
M. lenyanensis **sp. nov.**	Paratype	RMBH biv0628_6	54.8	35.0	18.6	n/a	n/a	n/a
M. lenyanensis **sp. nov.**	Paratype	RMBH biv0628_7	54.5	33.8	18.5	n/a	n/a	n/a
M. lenyanensis **sp. nov.**	Paratype	RMBH biv0628_8	38.1	24.5	12.6	n/a	n/a	n/a
M. lenyanensis **sp. nov.**	Paratype	RMBH biv0628_9	31.2	20.3	11.0	n/a	n/a	n/a
M. lenyanensis **sp. nov.**	Paratype	RMBH biv0628_10	39.9	26.3	12.9	n/a	n/a	n/a
M. mekongi **sp. nov.**	Holotype	RMBH biv0122	65.7	42.2	20.3	KX865861	KX865632	KX865733
Nyeinchanconcha nyeinchani **gen. & sp. nov.**	Holotype	NCSM 84884	50.9	27.8	15.6	KX822662	n/a	KX822618
Nyeinchanconcha nyeinchani **gen. & sp. nov.**	Paratype	NCSM 113351	33.7	15.1	7.9	n/a	n/a	n/a
Nyeinchanconcha nyeinchani **gen. & sp. nov.**	Paratype	UMMZ 304648	60.1	37.1	21.5	KP795025	KP795050	KP795008
Pseudodon kayinensis **sp. nov.**	Holotype	RMBH biv0618_1	59.6	34.9	17.7	MN275043	n/a	n/a
P. kayinensis **sp. nov.**	Paratype	RMBH biv0618_2	51.4	29.6	15.6	MN275044	n/a	n/a
P. kayinensis **sp. nov.**	Paratype	RMBH biv0618_5	37.8	22.4	10.3	n/a	n/a	n/a
P. kayinensis **sp. nov.**	Paratype	RMBH biv0637_1	47.0	29.2	14.1	MN275046	MN307241	MN307182
P. kayinensis **sp. nov.**	Paratype	RMBH biv0637_2	45.2	26.3	13.4	MN275047	n/a	n/a
P. kayinensis **sp. nov.**	Paratype	RMBH biv0637_3	53.0	29.1	16.1	MN275048	n/a	n/a
P. kayinensis **sp. nov.**	Paratype	RMBH biv0637_4	42.9	26.3	13.3	n/a	n/a	n/a
P. kayinensis **sp. nov.**	Paratype	RMBH biv0637_5	43.8	27.0	13.0	n/a	n/a	n/a
P. kayinensis **sp. nov.**	Paratype	RMBH biv0638_1	60.4	35.7	19.3	MN275049	MN307242	MN307183
P. kayinensis **sp. nov.**	Paratype	RMBH biv0638_2	66.7	37.4	21.1	MN275050	n/a	n/a
P. kayinensis **sp. nov.**	Paratype	RMBH biv0638_3	71.0	42.5	21.2	MN275051	n/a	n/a
Sundadontina brandti **sp. nov.**	Holotype	RMBH biv0475_2	85.4	53.7	27.7	MN275058	MN307249	MN307190
S. brandti **sp. nov.**	Paratype	RMBH biv0475_3	78.6	50.4	27.2	MN275059	MN307250	MN307191
S. brandti **sp. nov.**	Paratype	RMBH biv0475_4	71.3	43.8	24.1	MN275060	n/a	n/a
S. tanintharyiensis **sp. nov.**	Holotype	RMBH biv0643_4	57.1	40.8	23.2	MN275057	MN307248	MN307189
S. tanintharyiensis **sp. nov.**	Paratype	RMBH biv643_1	61.2	45.1	23.2	n/a	n/a	n/a
S. tanintharyiensis **sp. nov.**	Paratype	RMBH biv0643_6	39.2	27.2	14.7	n/a	n/a	n/a
S. taskaevi **sp. nov.**	Holotype	RMBH biv0475_1	82.7	52.2	29.3	MN275061	MN307251	MN307192
S. taskaevi **sp. nov.**	Paratype	RMBH biv0475_5	60.0	42.8	22.6	MN275062	n/a	n/a

*Type series of the new species are deposited in the Russian Museum of Biodiversity Hotspots [**RMBH**], Federal Center for Integrated Arctic Research, Russian Academy of Sciences, Arkhangelsk, Russia, North Carolina Museum of Natural Sciences [**NCSM**], Raleigh, United States of America, and the University of Michigan Museum of Zoology [**UMMZ**], Ann Arbor, United States of America. n/a – not available.

Supplementary Table 3. The most probable ancestral areas of the primary clades within freshwater mussels from Southeast Asia and India inferred from three different statistical modeling approaches. High support values (probability ≥70 %) are highlighted in bold

Clades	Ancestral areas	Type of biogeographic events	Probability of ancestral areas (%)		
			S-DIVA	Bayesian MCMC	Combined results
Pseudodontini	W. Indochina + Sundaland	Vicariance	**100.0**	28.0	64.0
Pseudodontina	W. Indochina	Intra-area radiation	**100.0**	**95.5**	**97.8**
Pilsbryoconchina	Sundaland	Intra-area radiation	**100.0**	**97.3**	**98.7**
The former subfamily Rectidentinae	Sundaland	Intra-area radiation	**100.0**	**96.9**	**98.4**
Rectidentini	Sundaland	Intra-area radiation	**100.0**	**99.8**	**99.9**
Contradentini	Sundaland	Dispersal	**100.0**	**97.6**	**98.8**
Yaukthwa + *Balwantia* + *Contradens*	W. Indochina + Sundaland	Vicariance	**100.0**	**88.8**	54.3
Yaukthwa + *Balwantia*	W. Indochina	Intra-area radiation	**100.0**	**91.6**	**95.8**
Contradens	Sundaland	Intra-area radiation	**100.0**	**99.5**	**99.8**
Trapezoideus	Sundaland	Intra-area radiation	**100.0**	**100.0**	**100.0**
Indochinellini	W. Indochina	Dispersal	**70.0**	**79.9**	**75.0**
Mekong's Indochinellini group (*Scabies* + *Scabiellus* + *Unionetta* + *Harmandia*) + *Radiatula*	W. Indochina + Sundaland	Vicariance	**100.0**	**91.2**	50.9
Mekong's Indochinellini group	Sundaland	Intra-area radiation	**100.0**	**90.2**	**95.1**
Radiatula	W. Indochina	Intra-area radiation	**100.0**	**99.9**	**100.0**
Indonaia	W. Indochina + India	Dispersal	**70.0**	45.0	47.0
Leoparreysiini + Parreysiini	W. Indochina + India	Vicariance	**100.0**	**78.4**	55.1
Leoparreysiini	W. Indochina	Intra-area radiation	**100.0**	**99.2**	**99.6**
Parreysiini	India	Intra-area radiation	**100.0**	**90.3**	**95.2**

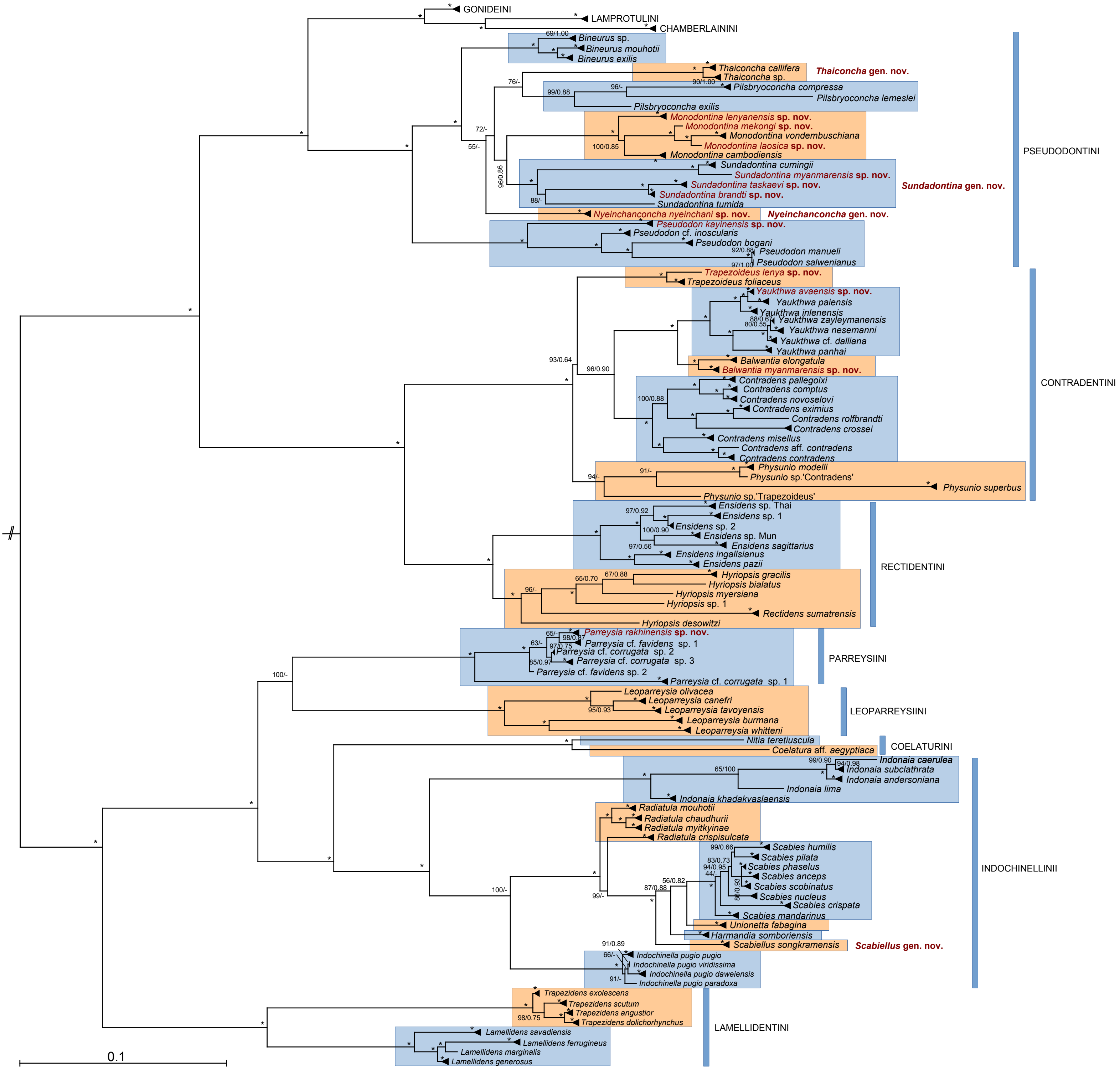

Supplementary Figure 1. Maximum likelihood phylogeny of the Parreysiinae and Gonideinae based on the complete data set of mitochondrial and nuclear sequences (five partitions: three codons of COI + 16S rRNA + 28S rRNA). Scale bar indicates the branch lengths. Black numbers near nodes are bootstrap support (BS) values of the IQ-TREE v1.6.11/BPP of MrBayes v3.2.6. Asterisks indicate high BS/BPP values ≥ 95/0.95. New generic and species names are colored red. Outgroup is not shown.
